# Treatment of Inborn Errors by Product Replacement: The Example of Inborn Errors of Bile Acid Synthesis

**DOI:** 10.1002/jimd.70081

**Published:** 2025-08-22

**Authors:** Peter T. Clayton, Rohit Hirachan, Elaine Murphy

**Affiliations:** ^1^ Inborn Errors of Metabolism, Genetics and Genomic Medicine UCL Great Ormond Street Institute of Child Health London UK; ^2^ Chemical Pathology Great Ormond Street Hospital for Children NHS Trust London UK; ^3^ Charles Dent Metabolic Unit National Hospital for Neurology and Neurosurgery London UK

**Keywords:** bile acid synthesis disorders, chenodeoxycholic acid, cholic acid, liver disease, neurological disorder, treatment

## Abstract

Many inborn errors of metabolism affect pathways involved in the synthesis of a metabolite that has an important biochemical or physiological function, and adverse effects of the disorder can be attributed to the lack of this metabolite. Thus, there is the opportunity for treatment by ‘product replacement’. One of the disorders in the pathways for the synthesis of bile acids from cholesterol, 3β‐hydroxy‐Δ5‐C27‐steroid dehydrogenase deficiency, causes cholestatic liver disease in infancy that can be treated very effectively with chenodeoxycholic acid (CDCA) and/or cholic acid (CA). There are several other enzyme deficiencies that can cause liver disease in infancy that improve with CDCA or CA or both (alongside a reduction of abnormal bile acids or alcohols); however, individuals with the same gene variant(s) may remain asymptomatic or have transient liver dysfunction that resolves spontaneously. In some disorders, the more usual presentation is with neurological disease later in childhood or in adolescence or adult life, for example, cerebrotendinous xanthomatosis (CTX), α‐methylacyl‐CoA racemase deficiency, and oxysterol 7α‐hydroxylase deficiency. Treatment with CDCA has been dramatically effective in the neurological disease of CTX. In the disorders of peroxisome biogenesis, liver disease is a part of the clinical picture although neurological symptoms tend to be predominant. Treatment with CDCA and CA (or CA alone) leads to a reduction in the levels of C27 bile acids. Some trials suggest this treatment leads to significant improvement in clinical status and liver function tests; others do not. Defects in individual peroxisomal enzymes and transporters vary in their clinical presentations. Treatment of acyl‐CoA oxidase 2 deficiency with ursodeoxycholic acid is discussed.

**Abbreviations**
3β‐HSDH: 3β‐hydroxysteroid‐Δ5‐C27‐steroid dehydrogenase/isomerase (encoded by *HSD3B7*)
5β‐reductase: Δ4‐3‐oxosteroid 5β‐reductase (encoded by *AKR1D1*)
ABCD3: ATP‐binding cassette family member D3 encoding peroxisomal membrane protein PMP70, the importer for C27 bile acid CoA esters
ACOX2: acyl‐CoA oxidase 2
AMACR: α‐methylacyl‐CoA racemase
BAAT: bile acid CoA; amino acid N‐acyl transferase
CA: cholic acid, 3α,7α,12α‐trihydroxy‐5‐cholanoic acid
CDCA: chenodeoxycholic acid. 3α,7α‐dihydroxy‐5β‐cholanoic acid
CTX: cerebrotendinous xanthomatosis
DBFP: D‐difunctional protein, peroxisomal enzyme catalysing enoyl‐CoA hydratase and 3‐hydroxyacyl‐CoA dehydrogenase reactions
DHCA: dihydroxycholestanoic acid, 3α,7α‐dihydroxy‐5β‐cholestanoic acid
FGF19: fibroblast growth factor 19
FGFR4: fibroblast growth factor receptor 4
FXR: farnesoid X receptor
HNF4: hepatocyte nuclear factor 4 (also called HNF4α, HNF4A)
LRH‐1: liver receptor homolog 1 (also called nuclear receptor subfamily 5, group A, member 2R [NR5A2])
ROS: reactive oxygen species
SCP2: sterol carrier protein 2 (peroxisomal thiolase 2, sterol‐carrier protein X [SCPX])
SHP: small heterodimer partner (also called nuclear receptor subfamily 0, group B, member 2 [NR0B2])
TGR5: Takeda G‐protein receptor 5
THCA: trihydroxycholestanoic acid, 3α,7α,12α‐trihydroxy‐5β‐cholestanoic acid
UDCA: ursodeoxycholic acid, 3α,7β‐dihydroxy‐5β‐cholanoic acid

## 1 Introduction

**FIGURE 1:**
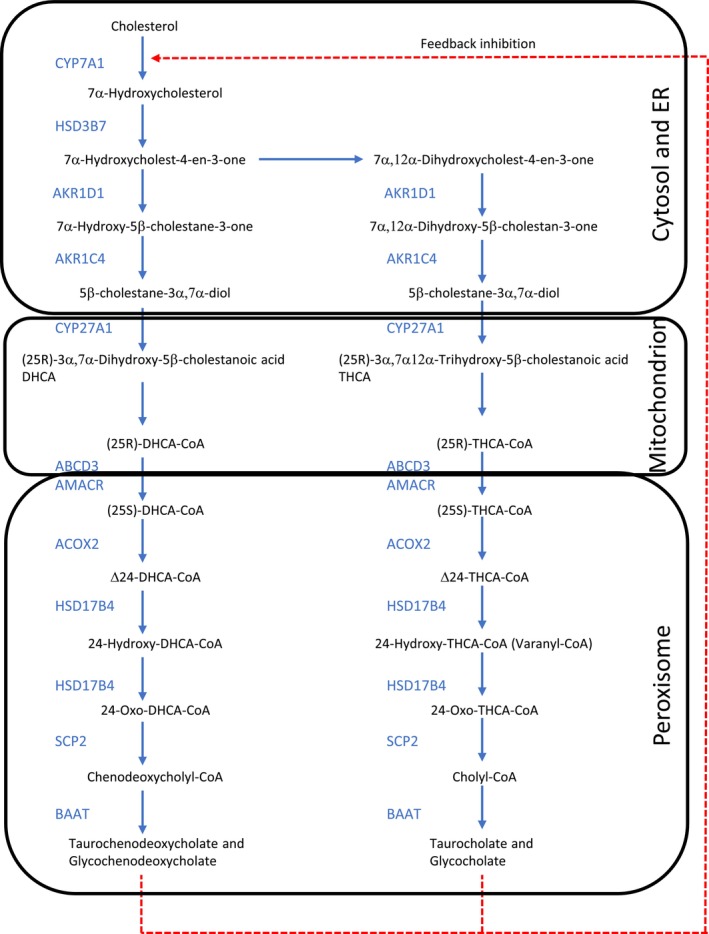
The ‘neutral pathway’ for bile acid synthesis. Enzymes catalysing the individual steps are indicated by the genes encoding them—see the table for the full enzyme name. In adults, the major pathway for the synthesis of CDCA and CA is the so‐called ‘neutral pathway’ that starts with the modification of the steroid nucleus of cholesterol by hydroxylation, isomerisation, and reduction reactions catalysed by enzymes located in the cytosol and endoplasmic reticulum (encoded by *CYP7A1*, *HSD3B7*, *AKR1D1*, and *AKR1C1/C4* for CDCA and additionally *CYP8B1* for CA). This is followed by side chain oxidation in the mitochondria, producing the C27 bile acids, 3α,7α‐dihydroxy‐5β‐cholestanoic acid (DHCA) and 3α,7α,12α‐trihydroxy‐5β‐cholestanoic acid (THCA). The 25R isomers of DHCA and THCA (as their CoA esters) are imported into the peroxisomes by the ABCD3 transporter (PMP70) and are converted to the 25S isomers by α‐methylacyl‐CoA racemase (*AMACR*). In the peroxisomes, oxidation of the side chain is completed by the β‐oxidation enzymes, acyl‐CoA oxidase 2 (*ACOX2*), the D‐bifunctional protein (*HSD17B4*) and the peroxisomal thiolase, SCPX (*SCP2*) [2]. The resulting CoA esters of CA and CDCA are converted to the glycine and taurine conjugates of the bile acids by the peroxisomal enzyme bile acid CoA; amino acid N‐acyl transferase [*BAAT*].

**FIGURE 2:**
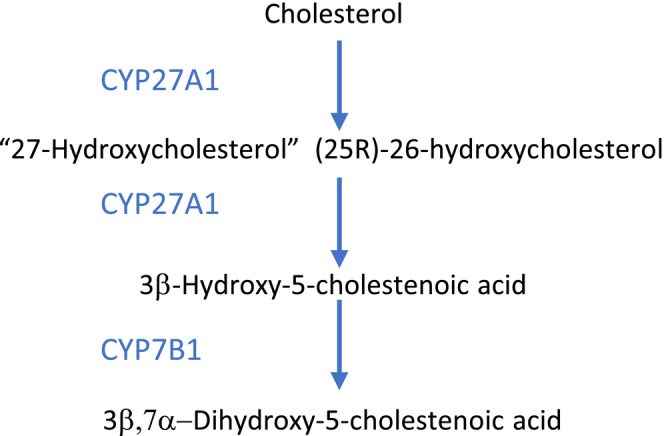
The first steps of the ‘acidic pathway’ for bile acid synthesis. Enzymes catalysing the individual steps are indicated by the genes encoding them—see the table for the full enzyme name. The acidic pathway starts with oxidation of the cholesterol side‐chain in the mitochondria, producing 27‐hydroxycholesterol and 3β‐hydroxy‐5‐cholestenoic acid. This is catalysed by the mitochondrial sterol 27‐hydroxylase encoded by *CYP27A1*. 3β‐Hydroxy‐5‐cholestenoic acid is then hydroxylated in the 7α‐position by the oxysterol 7α‐hydroxylase encoded by *CYP7B1*. Subsequent steps include (i) the completion of nuclear modifications which are thought to be catalysed by the same enzymes as in the neutral pathway (enzymes encoded by HSD*3B7*, *AKR1D1* and *AKR1C1/C4*), and (ii) beta‐oxidation in the peroxisomes (again using the same enzymes and transporter as in the neutral pathway), and finally conjugation to produce mainly the taurine and glycine conjugates of CDCA.

The major primary bile acids synthesised in the liver are the glycine and taurine conjugates of chenodeoxycholic acid (3α,7α‐dihydroxy‐5β‐cholanoic acid; CDCA) and cholic acid (3α,7α,12α‐trihydroxy‐5β‐cholanoic acid; CA) [1] (see Figure 1 for the neutral pathway, Figure 2 for the first few steps of the acidic bile pathway and Table 1 for a list of single enzyme/transporter defects affecting the pathways).

**TABLE 1:** Summary of the single enzyme/transporter disorders of bile acid synthesis, inheritance, clinical features, and potential treatment.

Condition	OMIM	Gene	Protein (and alternative names)	Inheritance	Clinical involvement			Treatment options
					Neuro	Hepatic	Other clinical features	
Cholesterol 7α‐hydroxylase Single‐nucleotide polymorphisms	118 455	*CYP7A1*	Cholesterol 7α‐hydroxylase	NA	No	No	Dyslipidaemia; premature CVD	Lipid lowering
3β‐Hydroxy‐Δ5‐C27‐steroid dehydrogenase deficiency	607 764	*HSD3B7*	3β‐hydroxy‐Δ5‐C27‐steroid oxidoreductase	AR	No	Yes		CDCA/CA (UDCA)
Δ4‐3‐Oxosteroid 5β‐reductase deficiency	604 741	*AKR1D1*	Aldo‐keto reductase family 1 member D1 Δ4‐3‐oxosteroid 5β‐reductase	AR	No	Yes		CDCA/CA (UDCA)
46,XY sex reversal (modifier of)	600 451	*AKR1C4*	Aldo‐keto reductase family 1 member C4 3α‐hydroxysteroid dehydrogenase type 1	AR	No	No	Disordered sexual development	None tried
Cerebrotendinous xanthomatosis	606 530	*CYP27A1*	Sterol 27‐hydroxylase	AR	Yes	Yes	Chronic diarrhoea; tendon xanthomas; cataracts, CVD	CDCA/CA
ABCD3 deficiency	170 995	*ABCD3*	ATP‐binding cassette, subfamily D, member 3	AR	No	Yes		None tried
Alpha‐methylacyl‐CoA racemase deficiency	604 489	*AMACR*	Alpha‐methylacyl‐CoA racemase	AR	Yes	Yes (rare)		Modified diet
Acyl‐CoA oxidase 2 deficiency	601 641	*ACOX2*	Acyl‐CoA oxidase 2, branched chain	AR	Yes	Yes		UDCA
D‐bifunctional protein deficiency	601 860	*HSD17B4*	17β‐hydroxysteroid dehydrogenase IV D‐bifunctional protein	AR	Yes	Yes	Hypogonadism	None tried
Oxysterol 7α‐hydroxylase deficiency	603 711	*CYP7B1*	Cytochrome P450, Family 7, Subfamily B, Polypeptide 1 Oxysterol 7α‐hydroxylase 1	AR	Yes (HSP 5A)	Yes		CDCA
SCPX thiolase deficiency	184 755	*SCP2*	Sterol carrier protein 2 Sterol carrier protein X	AR	Yes	No	Hypogonadism	None tried
Bile acid CoA: amino acid N‐acyl transferase deficiency	602 938	*BAAT*	Bile acid CoA: amino acid N‐acyl transferase	AR	No	Yes	Fat‐soluble vitamin deficiencies	Glycocholic acid/UDCA

Abbreviations: AR, autosomal recessive; CVD, cardiovascular disease; NA, not applicable; Neuro, neurological disease.

In adults, the first and main rate‐limiting step of the *neutral pathway for bile acid synthesis* is 7α‐hydroxylation of cholesterol. Cholesterol 7α‐hydroxylase (encoded by *CYP7A1*) is the site of feedback inhibition by CDCA and CA conjugates. This occurs by several mechanisms, including direct enzyme inhibition and down‐regulation of gene expression by two pathways [1]. In the liver, activation of FXR by bile acids induces SHP to inhibit HNF4 and LRH‐1 transactivation of *CYP7A1* and *CYP8B1* gene expression. Activation of FXR in the intestine induces FGF19, which is transported to the liver in the portal vein and activates the FGF receptor 4 (FGFR4)/β‐Klotho complex. This complex inhibits *CYP7A1* and *CYP8B1* gene expression.

The *acidic pathway for bile acid synthesis* can start outside the liver and be completed in the liver [1, 3, 4] (see Figure 2). The first steps produce oxysterols and cholestenoic acids, which are signalling molecules, so their signalling may be increased if they are before the block in the acidic pathway or decreased if they are after the block. In young infants, the acidic pathway is the main route for bile acid synthesis because *CYP7A1* is not expressed until the time of weaning.

The mechanism of negative feedback control of the acidic pathway is incompletely understood. The main rate‐limiting step is considered to be cholesterol transport to the inner mitochondrial membrane by steroidogenic acute regulatory protein (STARD1/StAR) [5].

Minor pathways for bile acid synthesis start with hydroxylations at C24 or C25. C24‐hydroxylation occurs in the brain catalysed by the *CYP46A1* gene product and C25‐hydroxylation occurs in macrophages catalysed by the *CH25H* gene product [1, 4, 6].

There are also additional pathways for bile acid synthesis of bile acids that can come into play when the major pathways are blocked. For example, in cerebrotendinous xanthomatosis, when the normal route of side‐chain oxidation is blocked by sterol 27‐hydroxylase deficiency, CA can be produced by microsomal hydroxylation at C24 and C25 (catalysed by CYP3A4 [7]) followed by cleavage between these carbon atoms [8].

The glycine and taurine conjugates of chenodeoxycholic acid and cholic bile acids are pumped into the biliary canaliculi by the bile salt export pump (BSEP encoded by *ABCB11*); this provides the driving force for the major component of bile flow (the bile acid dependent bile flow). Failure to synthesise adequate amounts of the primary bile acids can lead to a reduction in bile flow (cholestasis) with a build‐up in blood of compounds normally excreted in bile such as conjugated bilirubin. This should be correctable by product replacement (giving CDCA and/or CA). In the intestine, the glycine and taurine conjugated bile acids are powerful detergents that facilitate the micellar solubilisation of lipids, so if their production is impaired by an inborn error of bile acid synthesis, this can lead to the malabsorption of fats and the fat‐soluble vitamins (A, D, E and K).

## 2 Cerebrotendinous Xanthomatosis (CTX, *CYP27A1* Pathogenic Variants) (Figure S1)

CTX was the first bile acid synthesis defect to be treated by product replacement therapy. In 1968, Menkes et al. documented increased cholestanol in the CNS in CTX [9]. In 1971, Salen described the low concentration of CDCA in bile [10] and in 1974, Setoguchi et al. reported the substantial production of bile alcohols [11]. Berginer, Salen, and Shefer showed the efficacy of CDCA therapy in 1984 (see below) [12]. The underlying enzymatic defect was shown to be sterol 27‐hydroxylase deficiency in 1991 [13]. In the absence of sterol 27‐hydroxylase, the sterol side chain cannot be shortened by oxidation and 27‐carbon intermediates accumulate.

In 2016, a review of known cases of CTX indicated that the average age of diagnosis of CTX was 35 years, with a diagnostic delay of 16 years [14] and it was in 17 adults with CTX that Berginer demonstrated the clinical and biochemical efficacy of bile acid replacement therapy. So, this will be described before the paediatric presentations.

Berginer et al. reported that ‘before treatment, all subjects were symptomatic, with Achilles tendon xanthomas (15/17), cataracts (12/17), dementia (13/17), pyramidal‐tract signs (17/17), cerebellar dysfunction (13/17), mild peripheral neuropathy (7/17), electroencephalographic abnormalities (10/13), and abnormal cerebral computerised axial tomographic scans (10/12). After at least one year of CDCA treatment (750 mg per day), dementia cleared in 10 subjects, and pyramidal and cerebellar signs disappeared in 5 and improved in another 8. Peripheral neuropathy was no longer detected in six. The electroencephalogram became normal in five and showed fewer abnormalities in another three subjects. Cerebral computerised axial tomographic scans improved in seven patients; the changes included the disappearance of a cerebellar xanthoma in one case. Concomitantly, mean plasma cholestanol levels declined threefold, and abnormal bile acid synthesis was suppressed. We conclude that long‐term therapy with CDCA may correct the biochemical abnormalities and arrest and possibly reverse the progression of CTX’ [12]

CTX has been the subject of several recent reviews [14, 15, 16, 17, 18]; this paper will only consider the treatment by product replacement therapy and first treatment of adults, particularly their neuropsychiatric disease.

Intellectual disability may be present in the first decade of life, but neuropsychiatric dysfunction then becomes more obvious in late adolescence or early adult life. Psychiatric symptoms include behaviour disorders, agitation, hallucinations, and depression; dementia is common. Pyramidal signs (hyper‐reflexia and spasticity) and cerebellar signs (ataxia and dysarthria) are common. Extrapyramidal signs are present in some individuals, including parkinsonism, dystonia, myoclonus, and tremor. Non‐neurological features of CTX in adults include tendon xanthomata, premature atherosclerosis, diarrhoea, cataracts, and osteoporosis.

In 2018, Duell et al. reported on 43 individuals with CTX, with mean age at diagnosis 32 years [19]. They had the following major symptoms: cognitive impairment (74%), premature cataracts (70%), tendon xanthomata (77%) and neurological disease (81%). They were treated with CDCA 250 mg tds. Plasma cholestanol was normalised in 63%. Ninety‐one percent had normal liver function tests on treatment, but 9% developed moderate liver enzyme elevations (presumed CDCA hepatotoxicity) requiring reduction of the dose. Mean duration of follow‐up was 8 years (range 0–35 years). The treatment improved symptoms and then stabilised the disease in 57% of the cases; however, the disease continued to progress in 7 cases (20%). The latter were all aged 25 years or older, with significant neurological disease at diagnosis.

In 2019, Stelten et al. reported the largest retrospective cohort study of CDCA treatment (56 patients) [20]. Twenty‐four patients were started on treatment before the age of 24 years and 32 after the age of 24 years. The initial dose of CDCA ranged from 5 to 15 mg/kg/day in children < 16 years and up to 750 mg/day in adults. The dose of CDCA was adjusted on the basis of body weight and measurements of plasma cholestanol and urinary bile alcohol excretion. As a result of the biochemical measurement, the dose was increased to 1000 mg/day in nine patients. All patients who started on treatment before age 24 years had complete resolution of existing neurological symptoms and did not develop any new symptoms. In contrast, 61% of patients who started treatment after 24 years had neurological deterioration, with parkinsonism as the main treatment‐resistant feature. Rubio‐Agusti et al. had also reported on CDCA‐resistant atypical parkinsonism, with functional dopaminergic imaging frequently demonstrating presynaptic denervation [21]. It is possible that the product we should be replacing to treat atypical parkinsonism in CTX is not CDCA but 3β,7α‐dihydroxy‐5‐cholestenoic acid. The production of this LXR agonist is reduced in CTX [22] and LXR agonists can protect dopaminergic neurons [23].

Women with CTX have had successful pregnancies on CDCA (750 mg/day) [24]. In 11 pregnancies where mothers continued CDCA treatment, no complications were reported and babies were born at or near full term, with normal birthweight and Apgar scores. In eight pregnancies in which the mothers did not receive CDCA, two newborns had elevated bilirubin soon after birth. One woman who stopped CDCA during a pregnancy deteriorated neurologically while off treatment.

Compared to the extensive literature on the use of CDCA for the treatment of adults with CTX, there is only limited information on the use of CA. In 2019, Mandia et al. reported on 12 patients [25]. There were two subgroups: the treatment‐naïve group (who had never had CDCA prior to starting CA) and the non‐treatment‐naïve group who had CDCA prior to CA. They reported that treatment with CA significantly and strongly reduced cholestanol levels in all patients. Additionally, 10 out of 12 patients clinically improved or stabilised with CA treatment. Worsening was noted in one treatment‐naïve patient and one non‐treatment‐naïve patient, but both patients experienced similar outcomes with CDCA treatment. No adverse effects were reported from patients with CA treatment, whereas elevated transaminases were observed in some patients while they were treated with CDCA.

More than 25 years after CTX was identified as a neurological disease of adults, it was recognised as a cause of cholestatic liver disease in infancy. Indeed, the first case was initially thought not be CTX but another defect leading to increased urinary excretion of bile alcohol glucuronides (5β‐cholestane‐3α,7α,12α,24S,25‐pentol, 5β‐cholestane‐3α,7α,12α,25‐tetrol and 5β‐cholestane‐3α,7α,12α,24ξ‐tetrol) [26]. The 9‐week‐old boy had familial giant cell hepatitis and his affected sibling had died at the age of 13 months from progressive liver disease. Plasma concentrations of CDCA and CA were low. Treatment with CDCA (10 mg/kg/day) at age 19 weeks caused the plasma CDCA to rise to very high levels and there was a rise in bilirubin and in AST (to 2530 U/LU/L). The treatment was stopped but then tried again at 24 weeks with 10 mg/kg CDCA, then 5 mg/kg/day of both CDCA and CA and then CA alone at 5 mg/kg/day. On this second attempt at bile acid replacement therapy the plasma CDCA and CA rose to well above physiological levels but instead of a rise in bilirubin and AST there was a fall to normal values. He was well when last seen at the age of 20 years. In 2002, sequencing of *CYP27A* showed that he was homozygous for a deletion (525/526delG) causing a frameshift and a premature stop codon [27]. This genotype had been described in an adult female with classical symptoms of CTX (tendon xanthomata, cataracts and deteriorating cognitive function). A review of past medical histories of patients with CTX revealed that prolonged neonatal cholestatic jaundice was common. The family histories also revealed fetal and neonatal deaths among siblings of patients with CTX.

Other cases of CTX treated with CDCA or CA in the first years of life have been described. Pierre et al. described affected siblings [28]. The older sibling developed neonatal jaundice shortly after birth, and a biopsy showed chronic active hepatitis. Treatment at 3 months included ursodeoxycholic acid, and the abnormal liver function tests became normal by 8 months. He was subsequently shown to have CTX by *CYP27A* sequencing and the demonstration of urinary excretion of bile alcohol glucuronides. CA treatment (15 mg/kg/day) was commenced at age 14 months and led to marked reduction in bile alcohol excretion. The sibling was treated from 5 months and never developed jaundice. On follow‐up to 8 and 6.5 years, both siblings had low average neurodevelopmental performance, so it is not clear whether early treatment with cholic acid can prevent neurological damage.

Huidekoper et al. described a girl in whom CTX was diagnosed shortly after birth [29]. She was started on CDCA at (15 mg/kg/day). Within 6 weeks, she developed jaundice with hepatomegaly, so CDCA was stopped, after which liver size and function rapidly normalised. CDCA was then restarted and maintained at a dose of 5 mg/kg/day. On this regimen, cholestanol, liver enzymes, and total bilirubin have remained normal, and she has shown normal psychomotor development. So the current consensus is that infants diagnosed with CTX should be treated with 5 mg/kg/day of CDCA, and it may be necessary to reduce or stop if LFTs become abnormal.

Lipinski et al. reported on two siblings with neonatal cholestasis due to CTX [30]. The first developed cholestatic jaundice at 10 weeks and developed end‐stage liver disease. He was transplanted but died 3 years post‐transplant. *CYP27A* sequencing after he had died indicated the diagnosis of CTX. The second sibling had cholestatic jaundice from 1 day but was treated with CDCA (5 mg/kg/day) from 4 months of age. After a year of treatment, she was well with normal LFTs.

Lipinksi et al. reviewed the literature of infants diagnosed with CTX in the first 2 years of life up to 2021 [30]. Apart from the cases already described above, there were 5 infants who died without having received CDCA or CA, 2 who only received ursodeoxycholic acid (UDCA) but survived, one who received UDCA but required transplantation, 1 who was treated with CDCA but required transplantation, and one successfully treated with CDCA at a dose of 10–15 mg/kg/day.

It is clear that without bile acid replacement therapy, infants with CTX can have liver involvement that ranges from asymptomatic, through self‐limiting neonatal cholestasis to death from end stage liver disease/liver transplantation. Treatment with CDCA or CA can reduce bile alcohol excretion and normalise cholestanol and LFTs; however, high doses of CDCA can be hepatotoxic and a dose of 5 mg/kg/day is recommended.

In childhood, a common feature of CTX is diarrhoea. Treatment with CDCA (15 mg/kg/day up to 750 mg/day) [31] led to improvement in all patients; in 68% of cases, resolution was complete and sustained for as long as 25 years.

Stelten et al. have documented that autism is also a frequent early manifestation of CTX [32]; the behavioural problems stabilised or improved in children treated with CDCA.

## 3 3β‐Hydroxy‐Δ5‐C27‐Steroid Dehydrogenase Deficiency ( *HSD3B7* Pathogenic Variants) (Figure S2)

This disorder was identified in 1987 from the urine bile acids—3β,7α‐dihydroxy‐5‐cholenoic acid and 3β,7α,12α‐trihydroxy‐5‐cholenoic acid present as the sulphates and their glycine conjugates; the report suggested that the disorder should be treatable with bile acid replacement therapy [33], but the patient had been lost to follow‐up. Confirmation of the enzyme defect was achieved in 1990 [34]. The affected individual was located when he was aged 4 years and treated with CDCA, initially 18 mg/kg/day, to build up normal bile acid levels but reducing after 2 months to 9 mg/kg/day [35, 36]. This led to a very major reduction in the concentration of the unsaturated bile acids in the urine, and they were barely detectable in plasma. In bile, they had been completely replaced by the CDCA conjugated with glycine and taurine. The patient, who had been jaundiced with raised transaminases all his life, had normal liver function tests within weeks, and, on treatment, they have remained normal for 30 years. It should be noted, however, that at the start of treatment there was a transient rise in AST, consistent with hepatotoxicity of CDCA at a time when the BSEP was inhibited by high intrahepatic concentrations of the 3β‐hydroxy‐Δ5 bile acids.

The third patient we encountered presented a more difficult problem [37]. When assessed at the age of 7 months, her plasma bilirubin was 188 μmol/L, the AST was 300–760 U/L and a biopsy showed an aggressive hepatitis with hepatocyte necrosis and bridging fibrosis. Treatment was commenced with CDCA at 15 mg/kg/day when she was 9.6 months and, over the next 6 weeks, her bilirubin rose from 140 to 270 μmol/L and her AST rose from 270 to over 1000 U/LU/L. Treatment was then changed to CDCA 7 mg/kg/day plus CA 7 mg/kg/day. Over the next year her AST and bilirubin gradually normalised. She is well at the age of 36 years and had a successful pregnancy at the age of 32 remaining on the combination of CDCA and CA.

The UK experience of treating 13 patients presenting between 1989 and 2005 was reported in 2008 [38]. Five were treated with CA and CDCA (7 mg/kg/day of each), 7 with CDCA only (7–18 mg/kg/day) and one with CA only (8 mg/kg/day). There was quite a variation in the time taken to normalisation of LFTs: bilirubin median 3 months, range 1–16 months; AST median 2.5 months, range 1–45 months. However, after a median follow‐up of 5.5 years (range 1–17 years) 12/13 showed no signs of liver disease or fat‐soluble vitamin deficiency; 1/13 had been lost to follow‐up. Four patients who had pre‐ and post‐treatment biopsies showed improved liver histology. In addition to the treated patients, there was documentation of three children who were not treated and died before the age of 5 years.

In 2009, Gonzalez et al. reported 13 patients with 3β‐HSDH deficiency who were treated with CA alone [39]. Treatment was started at a median age of 3.9 years (range 0.3–13.1 years) with a median duration of follow‐up of 12.4 years (range 5.6–15 years). The mean dose of CA was 13 mg/kg/day at initiation of treatment and 6 mg/kg/day at the most recent evaluation. Physical examination findings, laboratory tests, and abdominal ultrasound all normalised on treatment. The urinary excretion of 3β‐hydroxy‐Δ5 bile acids was reduced 500‐fold. Liver biopsies performed after at least 5 years of CA therapy showed marked improvement. Four successful pregnancies were documented in two patients with 3β‐HSDH deficiency who took CA throughout pregnancy. There were transient signs of CA overdose in 4 children. Clinical features of toxicity included pruritus, diarrhoea, and elevation of GGT, ALT, and total serum bile acids, which resolved with reduction of the dose of CA. Gonzales et al. observed that the suppression of excretion of 3β‐hydroxy‐Δ5 bile acid excretion was much greater with CA than with UDCA, consistent with the inability of UDCA to inhibit oxysterol 7α‐hydroxylase.

In 2010, Riello et al. described the treatment of 5 patients with 3β‐HSDH deficiency [40]. Three had presented with liver disease (giant cell hepatitis [1], biliary cirrhosis [1] and cryptogenic cirrhosis [1]) and two were detected by neonatal screening. Treatment was with UDCA and CDCA. The dose was adjusted to maintain urinary excretion of 3β,7α‐dihydroxy‐5‐cholenoic acid at < 20 μmol/mmol creatinine; this was achieved after 3–28 months of treatment, and in some patients, doses could be reduced to 5 mg/kg/day of both bile acids. All patients normalised their liver function tests.

Our experience has been similar to that described by Gonzales et al. and Riello et al.: urine bile acid monitoring to demonstrate low levels of excretion of unsaturated bile acids works well for monitoring adequate inhibition of cholesterol 7α‐hydroxylase, but monitoring the saturated bile acids in blood is the best way to show that the dose of CDCA or CA is too high. In 2016, we reported a method for quantitation of both 3β‐hydroxy‐Δ5 bile acids and saturated bile acids (from treatment) in dried blood (or plasma) spots [41]. This makes sampling easier and transport from a distant hospital much easier. We used this method to monitor the early days of treatment of three newly diagnosed children with 3β‐HSDH deficiency [42]. Patient 1 was treated with CA 15 mg/kg/day from day 1. Patients 2 and 3 were treated with the following graded introduction of CA after priming with ursodeoxycholic acid (UDCA): Week 1: 7.5 mg/kg/day UDCA bd; Week 2: 7.5 mg/kg UDCA am and 5 mg/kg CA pm; Week 3: 7.5 mg/kg UDCA am, 5 mg/kg CA midday, and nocte; Week 4: 5 mg/kg CA tds. On CA treatment, all patients showed complete resolution of cholestatic liver disease, malabsorption of fat and fat‐soluble vitamins, and a marked reduction of urinary excretion of unsaturated bile acids. Patient 1, treated with CA at a dose of 15 mg/kg/day, showed a transient rise in the concentrations of TCA and GCA in the first few days of treatment. The levels reached in blood (TCA = 24 μM; control range ≤ 1 μM; and GCA = 83 μM; control range ≤ 3 μM) are levels similar to those seen in bile duct obstruction and could indicate TCA and GCA levels in the hepatocyte that are toxic. The highest recorded transaminase levels (AST 868 U/L; ALT 680 U/L) were measured on the day of starting treatment. The total bilirubin increased from 24 mg/dL to 28 mg/dL over the first 4 days of treatment. Transaminases remained > 100 U/L for 2 months and took 9 months to normalise. In patients 2 and 3, levels of TCA and GCA did not rise above the normal range, and transaminases did not rise. We conclude that inducing choleresis with UDCA and then starting CA at a low dose and building up slowly may avoid the early CA toxicity.

In 2010, Nittono et al. reported a good response to treatment and two successful pregnancies in a Japanese patient taking a combination of CDCA (7.4 mg/kg/day) and CA (2.2 mg/kg/day) [43].

In 2017, Heubi, Bove, and Setchell reported the outcome of a clinical trial of CA (10–15 mg/kg/day in a cohort of 70 individuals, 50 with single enzyme defects), over 30 of whom had 3β‐HSDH deficiency [44]. The cohort as a whole showed a significant reduction in urinary excretion of abnormal cholanoids and significant reductions of serum aspartate aminotransferase and alanine aminotransferase. There were 6 serious adverse effects in the single enzyme defect group, including death due to disease progression in one patient with 3β‐HSDH deficiency.

Kimura et al. have reported 3 Japanese patients with 3β‐HSDH deficiency who have received treatment with CDCA alone for 10 to 21 years [45]. Doses were 4.0–7.8 mg/kg/day and the authors reported gradual improvement in liver function tests with normal results at the most recent follow‐up.

In 2022, Zhang et al. conducted a systematic review of the literature on 3β‐HSDH deficiency [46] and reported that, of the patients identified, 44 had been treated with CDCA alone, 17 with CA alone, 8 with UDCA plus CA, 7 with CDCA plus CA, and one with UDCA alone. 81.6% survived on bile acid treatment, but 18.4% died or required a liver transplant. Age at presentation appeared to be an important determinant of prognosis, with worse outcomes if the age at presentation was < 1 years.

There are reports of adults with liver disease due to 3β‐HSDH deficiency. Kobayashi et al. described a Japanese woman with jaundice and bleeding due to vitamin K deficiency during childhood, who then presented again at the age of 23 with cholestatic liver disease [47]. Fischler et al. described a patient whose two older siblings had died from progressive cholestatic liver disease and who developed neonatal cholestasis and rickets but recovered during childhood [48]. The patient presented again at 26 years of age with jaundice and pathological liver function tests which normalised with UDCA treatment. From these reports, we can conclude that the risk of serious liver disease from 3β‐HSDH deficiency persists from infancy into adult life.

### 3.1 Conclusion

3β‐HSDH deficiency can cause liver disease and malabsorption of fat and fat‐soluble vitamins at any age; without treatment, the liver disease can progress to end stage and death or transplantation. Treatment with either CDCA or CA or both can suppress the production of 3β,7α‐dihydroxy‐5‐cholenoic acid and 3β,7α,12α‐trihydroxy‐5‐cholenoic acid and can lead to normalisation of LFTs, improvement in the liver biopsy appearance, and long‐term survival. However, particularly at the start of treatment when the BSEP is inhibited by the unsaturated bile acids, blood levels of conjugates of CDCA and/or CA can rise to substantially above normal, and the transaminases and bilirubin can rise, indicating hepatotoxicity. UDCA is not as effective as CDCA or CA at suppressing the production of 3β,7α‐dihydroxy‐5‐cholenoic acid and 3β,7α,12α‐trihydroxy‐5‐cholenoic acid but can ameliorate liver disease in the short term. UDCA has potent choleretic properties due to cholehepatic cycling, and this may generate some bile flow even when the BSEP is inhibited by the unsaturated bile acids. It may be useful to start treatment with UDCA and then cross over to CDCA or CA to avoid hepatotoxicity of CDCA or CA.

## 4 Δ4‐3‐Oxosteroid 5β‐Reductase Deficiency ( *AKR1D1* Pathogenic Variants) (Figure S3)

In 1988, Setchell et al. described twins with neonatal hepatitis in whom the major urinary cholanoids were the taurine conjugates of 7α‐hydroxy‐3‐oxo‐4‐cholenoic acid and 7α,12α‐dihydroxy‐3‐oxo‐4‐cholenoic acid, indicating reduced activity of Δ4‐3‐oxosteroid 5β‐reductase [49].

When the specificity of excretion of the 3‐oxo‐Δ4 bile acids was tested, it became clear that increased excretion of these unsaturated bile acids can occur in liver disease in childhood with a wide range of aetiologies including virus infections, autoimmune hepatitis, biliary atresia, and tyrosinaemia (fumaryl acetoacetase deficiency) [50]. It has been known since 1998 that the 5β‐reductase enzyme is vulnerable to aldehyde stress [51]. More recently, it has been shown that, in vitro in HepG2 cells and in vivo in mice, the expression of the 5β‐reductase gene (*AKR1D1*) is repressed by CDCA and upregulated by CA [52]. Thus, there is the potential for reduced activity of 5β‐reductase in individuals with a high CDCA to CA ratio in the hepatocytes, and this is something that occurs in many severe liver diseases.

In 2003 it was shown that true primary 5β‐reductase deficiency could be caused by biallelic pathogenic variants in *AKR1D1* [53]. Of the three patients with confirmed biallelic mutations in *AKR1D1*, one had improved on a combination of CDCA and CA (both at a dose of 8 mg/kg/day), having failed to show any improvement on UDCA (12–20 mg/kg/day). The second showed initial improvement on CDCA + CA (8 mg/kg/day of both) but then deteriorated with a high CDCA concentration (128 μmol/L). CDCA was stopped and then restarted, but LFTs continued to deteriorate in association with a positive PCR test for cytomegalovirus, and he was referred for transplantation. The third infant with biallelic *AKR1D1* mutations failed to respond significantly to UDCA (30 mg bd) or CDCA (10 mg tds) and was referred for transplantation at 19 weeks. The first patient, whose liver function returned to normal following treatment with CDCA and CA, was reviewed at the age of 13 years. Despite having stopped the bile acid replacement therapy, she was well with normal liver function.

In 2009, Gonzales et al. reported two infants with primary 5β‐reductase deficiency (proven by *AKR1D1* sequencing) who were treated with CA starting at 13 mg/kg/day but gradually reducing to 4.8–5.7 mg/kg/day [39]. The CA treatment led to a 30‐fold reduction in the excretion of 3‐oxo‐Δ4 bile acids. Prior to CA treatment, liver histology showed severe inflammation with intense cholestasis and marked fibrosis. After 14 months of treatment with a combination of CA and UDCA, there was a reduction in inflammation and cholestasis but not in fibrosis. In one patient, it was possible to wean on to CA monotherapy; but one needed to continue with UDCA at a dose of 4 mg/kg/day given separately from the CA. This small amount of UDCA was necessary to maintain normal LFTs; without it, the ALT was 1.5× normal.

In the large 2017 clinical trial of CA (mentioned above), four of the patients with 5β‐reductase deficiency had end‐stage liver disease at the time of starting CA therapy and died despite the treatment [44].

In 2019, Zhang et al. reported the follow‐up of treatment of 12 patients in whom the diagnosis of 5β‐reductase deficiency had been confirmed by urine bile acid analysis and by sequencing of *AKR1D1* [54]. Nine of the 12 were treated with UDCA prior to the diagnosis of 5β‐reductase deficiency; 3 of these received high‐dose UDCA (30–40 mg/kg/day) the other 6 received 10–20 mg/kg/day. Complete normalisation of LFTs was only seen in one patient; in the majority, LFTs remained abnormal. Eleven patients received CDCA at an initial dose of 5–12 mg/kg/day. Five had to temporarily stop or have the dose reduced because of a marked elevation of levels of serum ALT, AST, and total bile acid attributed to CDCA hepatotoxicity. In 6, there was steady improvement. When their jaundice had resolved, some patients needed an increase in dose to maintain suppression of urinary excretion of 3‐oxo‐Δ4 bile acids. The final CDCA dose was 5.5–10 mg/kg/day. All 11 were well after a follow‐up of 0.5–6.4 years. The 12th patient continued to deteriorate despite CDCA treatment and underwent liver transplantation.

In 2020, Chen et al. described two patients with 5β‐reductase deficiency (proven *AKR1D1* mutations) who were successfully treated with cholic acid (7.5 mg/kg/day) and had normal liver function tests after 20‐year follow‐up [55].

In 2023, Gardin et al. reported the outcome of CA treatment in 16 patients with 5β‐reductase deficiency, all confirmed by *AKR1D1* sequencing [56]. Median age at presentation was 2 months. Fourteen had had cholestatic jaundice; three had severe bleeding. Fifteen patients were treated with UDCA before diagnosis, with partial improvement in eight. All 16 were treated with CA from a median age of 8.1 months (range 3.1–159 months); LFTs normalised and there was a 12‐fold decrease in the urinary excretion of 3‐oxo‐Δ4 bile acids. After a median duration of CA therapy of 4.5 years (range 1.1–24) all patients were alive with their native liver.

The retrospective studies of Zhang et al. and Gardin et al. show clearly that when patients with biallelic mutations in *AKR1D1* develop liver disease, the liver disease improves with CDCA or CA treatment, but do all individuals with pathogenic mutations in *AKR1D1* and a urine bile acid profile indicating that 3‐oxo‐Δ4 bile acids are the major cholanoids in the urine need treatment? Morgan et al. described a family with mutations in *AKR1D1* (c.587delG) and we have followed up this family with Prof Deirdre Kelly [57, 58]. Four siblings and a cousin were homozygous for c.587delG. The cousin and one sibling died of progressive liver disease; another sibling presented with cholestatic liver disease and improved with cholic acid, but two further siblings who had a urine bile acid profile showing marked excretion of 3‐oxo‐Δ4 bile acids have remained asymptomatic with normal LFTs. In a family from Norway, a 37‐year‐old lady with learning difficulties was found to be homozygous for a pathogenic variant in *AKR1D1*. Her urine bile acid spectrum was typical of 5β‐reductase deficiency. She has never had any symptoms of liver disease and has normal LFTs; however, a brother born in 1978 died of progressive liver disease in infancy of unknown cause. Kimura et al. described a Japanese girl with *AKR1D1* mutations who only ever received UDCA for cholestasis in infancy and has remained well after that was discontinued [59]. They also described three Vietnamese siblings with *AKR1D1* mutations; the propositus had cholestasis from birth and did well on CDCA treatment, but an 8‐year‐old sister and 10‐year‐old brother have never been jaundiced or shown abnormality of LFTs.

### 4.1 Conclusion

In 2025, the diagnosis of Δ4‐3‐oxosteroid 5β‐reductase deficiency requires both the demonstration that the major bile acids in the urine are conjugates of 7α‐hydroxy‐3‐oxo‐4‐cholenoic acid and 7α,12α‐dihydroxy‐3‐oxo‐4cholenoic acid and the demonstration of biallelic pathogenic mutations in *AKR1D1*.

Patients with cholestatic liver disease may show some improvement with UDCA, but primary bile acid replacement (CA and/or CDCA) is required to reduce the production of the unsaturated bile acids that inhibit the BSEP. Treatment with both CA and CDCA has produced good long‐term results. Monitoring of urine bile acids shows a marked reduction in the excretion of 3‐oxo‐Δ4 bile acids. Measurement of LFTs and plasma CDCA and CA levels is important to avoid hepatotoxicity of CDCA or CA.

## 5 Oxysterol 7α‐Hydroxylase Deficiency ( *CYP7B1* Pathogenic Variants (Figure S4)

In 1998, Setchell et al. described a 10‐week‐old boy who presented with severe cholestasis, cirrhosis, and liver synthetic failure and whose urine spectrum indicated that the major cholanoid peaks were attributable to 3β‐sulfooxy‐5‐cholenic acid and its glycine conjugate [60]. Reduced activity of oxysterol 7α‐hydroxylase and a homozygous pathogenic variant in *CYP7B1* were demonstrated. Treatment with UDCA (15 mg/kg/day) resulted in a marked increase in bilirubin and transaminases and was discontinued. Treatment with CA (15 mg/kg/day) produced no clinical improvement, and albumin continued to fall and prothrombin time to increase, so CA treatment was discontinued after 49 days. He underwent transplantation at 4.5 months.

In 2008, Ueki et al. described an infant with jaundice from the neonatal period and worsening up to 5 months [61]. LFTs were severely deranged, and a biopsy showed cirrhosis with bile duct proliferation, lobular disarray, and giant cell transformation. Treatment with UDCA did not lower the bilirubin or transaminases. Liver function deteriorated in association with an episode of infection, and he died at 11 months.

Also in 2008, Tsaousidou et al. showed that mutations in *CYP7B1* cause a recessive form of hereditary spastic paraparesis (HSP5/SPG5) [62]. Affected individuals present between 1 year and 41 years with weakness of the legs, with hypertonia and hyper‐reflexia, and posterior column sensory impairment with reduced vibration sensing and joint position sensing, as well as some bladder dysfunction.

In 2014, Dai et al. described an infant who presented with liver failure and hypoglycaemia at 3 months. The liver disease had been steadily worsening on UDCA [63]. Treatment with CDCA (15 mg/kg/day) led to normalisation of LFTs, and a drop in the urinary excretion of the sulphate and taurine conjugate of 3β‐hydroxy‐5‐cholenoic acid (a hepatotoxic bile acid). However, a liver biopsy taken 2 weeks after starting CDCA showed that during the period of active liver disease, he had developed advanced fibrosis with a micronodular cirrhosis pattern. The lobules showed features of a giant cell hepatitis with lobular disarray and liver cell rosettes. On follow‐up he had no symptoms or signs of liver disease, but by the age of 13.5 years he had clear signs of spastic diplegia and was getting very tired after 10–15 min walking.

In 2017, Schöls et al. studied 34 individuals with proven biallelic mutations in *CYP7B1* [64]. Presentation was usually in childhood or adolescence (median 13 years). Gait ataxia was a common early symptom. Affected individuals lost the ability to walk independently after a median disease duration of 23 years and became wheelchair dependent after a median 33 years. There was a correlation between serum 27‐hydroxycholesterol and disease severity. Treatment with Atorvastatin for 9 weeks reduced serum 27‐hydroxycholesterol and 25‐hydroxycholesterol; but, as expected with this short‐term trial, there were no effects on clinical parameters.

In 2021, Tang et al. described a 5‐month‐old infant with progressive cholestasis and prolonged prothrombin time from 1 months [65]. There had been no response to UDCA. She was then treated with CDCA (6 mg/kg/day). Her liver function rapidly improved, urine atypical bile acids normalised, and she remained well up to the last follow‐up at 23 months. Her 15‐year‐old brother, with no history of infantile cholestasis but harbouring the same mutations in *CYP7B1*, had gait abnormality from 13 years of age. Neurological examination revealed hyperreflexia and spasticity of the lower limbs.

In 2022, Mo et al. reported an infant ‘successfully treated with UDCA’; however, normal values of bilirubin and transaminases were never achieved [66].

In 2024, Wang et al. reported two infants who presented with neonatal cholestasis and hepatomegaly [67]. The first infant responded to CDCA treatment with normalisation of LFTs; but the second required liver transplantation.

### 5.1 Conclusion

Biallelic *CYP7B1* mutations can cause severe cholestatic liver disease in infancy which can lead to death or the requirement for transplantation but which can be successfully treated with CDCA. On the other hand, the same mutations (even in a sibling) might cause no detected liver dysfunction and present later in childhood or in adulthood with progressive spastic paraparesis.

There is no evidence that CDCA treatment can prevent the spastic paraparesis caused by biallelic *CYP7B1* mutations, although in induced pluripotent stem cell‐derived neurons from patients, CYP7B1 deficiency leads to impaired neurofilament expression as well as axonal degeneration, which could be rescued with CDCA [68]. Perhaps other product replacement therapies should be considered for the spastic paraparesis; deficiency of 3β,7α‐dihydroxy‐5‐cholestenoic acid may contribute to motor neuron loss [22].

## 6 Single Peroxisomal Enzyme/Transporter Deficiencies

In the neutral pathway for bile acid synthesis, mitochondrial oxidation of 5β‐cholestane‐3α,7α‐diol and 5β‐cholestane‐3α,7α,12α‐triol produces the 25R isomers of DHCA and THCA. After conversion to the CoA esters, these C27 bile acids need to be converted to the 25S isomers and undergo β‐oxidation in the peroxisomes. Defects in these steps lead to the build‐up of DHCA and THCA. Conjugated and unconjugated C27 bile acids can impair mitochondrial respiratory chain function and increase the accumulation of reactive oxygen species (ROS) leading to cell death in rat hepatoma cells [69]. Wanders and Ferdinandusse make the case that it is the deficiencies of peroxisomal single enzyme defects that lead to the accumulation of DHCA and THCA that have liver disease [70]. The glycine and taurine conjugates of DHCA and THCA can be secreted into bile and are not inhibitors of the BSEP. However, DHCA‐CoA and THCA‐CoA are poor substrates for BAAT, and if they accumulate in the cytosol rather than in the peroxisome, they may not have access to BAAT. They will then be converted by thioesterases to unconjugated DHCA and THCA, and these are poor substrates for the BSEP and are likely to cause hepatotoxicity.

Here, we will consider first the defects of single peroxisomal enzymes/transporters involved in DHCA and THCA metabolism and then the defects of peroxisome biogenesis (Zellweger spectrum disorders). When considering the role of bile acid replacement therapy, it is important to consider not only the liver disease but also the neurological disease which may present later in life.

## 7 α‐Methyl‐Acyl‐CoA Racemase Deficiency ( *AMACR* Pathogenic Variants) (Figure S5)

The first individuals with mutations in *AMACR* were described in 2000 [71]. Two suffered from adult‐onset sensory motor neuropathy. One patient also had pigmentary retinopathy, whereas the other patient had upper motor neuron signs in the legs. The third patient was a child with no neuropathy but with liver disease: it was uncertain how much AMACR deficiency was contributing to cholestatic liver disease as the child also had Niemann‐Pick C disease. The first neonatal presentation was described by Van Veldhoven et al. (2001) [72] and Setchell et al. (2003) [73]. This was a 2‐week‐old African‐American girl who presented with coagulopathy, vitamin D and E deficiencies, and mild cholestasis. The urine bile acid spectrum at 7 weeks showed a major excretion of taurotetrahydroxycholestanoic acids while the major bile acid in bile was (25R)‐3α,7α,12α‐trihydroxycholestanoic acid (THCA). The liver biopsy showed giant cell transformation and scattered necrotic eosinophilic hepatocytes. The patient was treated with CA at a dose of 15 mg/kg/day which was associated with a total suppression of the urinary excretion of taurotetrahydroxycholestanoic acids, normalisation of LFTs, and normal fat‐soluble vitamin levels without supplementation. The patient was well at the age of 7 years.

A 2024 review of the literature of the 24 confirmed cases of AMACR deficiency revealed that there were only four individuals whose presentation included liver dysfunction in infancy or childhood and three whose presentation included cholelithiasis in the teens/early twenties [74]. The more common presentations were with neurological disease in adulthood. The neurological features varied considerably but included developmental delay, seizures, sensory motor neuropathy, relapsing encephalopathy, pigmentary retinopathy, optic atrophy, headache, migraine, depression, bipolar disorder, tremor, transient ischaemic attack, hemiparesis, homonymous hemianopia, schizophrenia, neuroleptic malignant syndrome, rhabdomyolysis, hemineglect syndrome, and central apnoea.

AMACR deficiency impairs metabolism of pristanic acid, and hence phytanic acid, and so patients have been treated with a low phytanic acid diet. Improvement in neurological symptoms has been observed in some but not others. One patient with hepatic dysfunction in infancy improved with a diet low in phytanic acid and pristanic acid. There are no reports of treatment of neurological disease with CA or CDCA.

### 7.1 Conclusion

Like CTX, AMACR deficiency can present with cholestasis and fat‐soluble vitamin deficiency in infancy but more often presents later in childhood or in adult life with neurological dysfunction. Thus, if there is neonatal liver dysfunction in the latter group, it is subclinical and self‐limiting. CA treatment can probably speed resolution of clinically presenting liver disease/fat‐soluble vitamin deficiency. There is no information available on whether bile acid replacement therapy can ameliorate neurological disease.

## 8 Acyl‐CoA Oxidase 2 Deficiency ( *ACOX2* Pathogenic Variants) (Figure S6)

In 2017, Vilarinho et al. described a homozygous pathogenic variant (c.207T>A; p.Y69*) in *ACOX2* causing ACOX2 deficiency in an 8‐year‐old boy with intermittently elevated transaminase levels, liver fibrosis, mild ataxia, and cognitive impairment [75]. He was treated with CA for a year (unpublished observations of authors listed in ref. [75]). Unfortunately, the liver function tests had normalised prior to starting treatment; thus, they were not useful for assessing efficacy. The levels of unconjugated DHCA and THCA came down with treatment; the levels of the conjugated C27 bile acids were variable, with some values still elevated. There was no improvement in the neurological findings of tremor, mild dysmetria, and mild cognitive impairment.

Also in 2017, Monte et al. described a 16‐year‐old with unexplained transaminaemia (2–5× upper limit of normal) 2 years following a transient episode of acute ibuprofen/diclofenac‐induced hepatitis [76]. The transaminases reverted to normal when he was treated with cholestyramine, and Monte et al. argued that this suggested that the hepatotoxicity was due to C27 bile acids, and this was reduced by the bile acid sequestering resin. They showed that THCA induced, in HepG2 cells, a dose‐related increase in ROS production with concomitant reduction of cell viability, and that this could be efficiently prevented by overexpressing wild type *ACOX2* but not the patients mutated *ACOX2 (*c.673C>T; p.R225W).

In 2018, Ferdinandusse et al. described a child with a 4 nucleotide deletion in *ACOX2* causing a frame shift and premature stop (c.462–464delTCTG; p.Thr154Serfs*25) [77]. This child had a severe phenotype, requiring resuscitation at birth and presenting with multiple congenital malformations, electrolyte abnormalities, a neurogenic myopathy, and persistent elevation of alanine aminotransferase. She died at 6 months. Bile acid replacement was not attempted.

Further investigation of the R225W variant in the Spanish population indicated an allele frequency of 0.7%; this led to a search for cases of ACOX2 deficiency in individuals with raised transaminases [78]. In 33 patients with persistent hypertransaminaemia, Alonso‐Pena et al. identified 4 individuals with raised plasma C27 bile acids, and further investigation of the 13 members of the families of these individuals revealed a further 3 cases. Their first case was treated empirically, before diagnosis, with UDCA. After 3 weeks, plasma transaminases had dropped to normal values, and the patient's main symptom of fatigue was improved. He was then shown to have increased concentrations of C27 bile acids, and genetic analysis revealed that he was homozygous for R225W. Investigation of his family showed that his mother, brother, and uncle were all homozygous for R225W and had increased C27 bile acids in serum. All 3 had undergone a cholecystectomy for complicated cholelithiasis. The uncle was found to have raised transaminases, and he was treated with ursodeoxycholic acid (UDCA) which normalised the transaminases but not the abnormal proportion of C27 bile acids in serum. All the patients identified by Alonso‐Pena et al. (whether homozygous for R225W or compound heterozygous for R225W and c.456–459del) showed a reduction in hypertransaminaemia with UDCA treatment (12—15 mg/kg/day) but there was no consistent reduction in the proportion of 27 bile acids in the serum bile acid profile.

In the above studies of ACOX2 deficiency, the intermediates that were measured were DHCA and THCA, cholestanoic acids derived from intermediates in the neutral pathway. What happens to the acidic pathway? Yutuc et al. used a sensitive method for the detection of compounds with a 3β‐hydroxy‐Δ5 or 3‐oxo‐Δ4 structure to study the unsaturated C27 bile acids in the patient described by Vilarinho et al. [75, 79]. The plasma concentration of 3β,7α‐dihydroxy‐5‐cholestenoic acid was 314 mg/mL compared to the control value of < 45 ng/mL, and the concentration of 7α‐hydroxy‐3‐oxo‐4‐cholestenoic acid was 487 ng/mL (control < 105 ng/mL). The relevance to the liver disease is that bile acids with a 3β‐hydroxy‐Δ5‐ or 3‐oxo‐Δ4 structure can inhibit the BSEP [80]. This could contribute to hepatotoxicity alongside the effects of THCA and DHCA described above. The study by Yutuc et al. also showed that in ACOX2 deficiency, there is accumulation of 3β,7β‐dihydroxy‐5‐cholestenoic acid, suggesting that the acidic pathway may be a route for the synthesis of UDCA. UDCA is normally considered a secondary or tertiary bile acid synthesised through the action of intestinal bacteria [81].

### 8.1 Conclusion

In ACOX2 deficiency there is evidence that liver disease may be improved by treatment with UDCA. UDCA does not inhibit the rate‐limiting steps in bile acid synthesis but has unique choleretic properties that may improve bile flow when the production of conjugates of CDCA and CA is reduced.

## 9 PMP 70 Peroxisomal Transporter Deficiency ( *ABCD3* Pathogenic Variants)

Mutations in *ABCD3* encoding PMP70 cause a defect of the peroxisomal import of THCA‐CoA, DHCA‐CoA, and branched chain fatty acyl‐CoAs. The first described patient presented with hepatosplenomegaly and severe liver dysfunction [82]. Developmental milestones were normal. Plasma concentrations of DHCA and THCA were very high. The liver disease progressed, and she required a liver transplant at the age of 4 years.

## 10 D‐Bifunctional Protein Deficiency ( *HSD17B4* Pathogenic Variants) (Figures S7 and S8)

In D‐bifunctional protein deficiency, there is a defect in the β‐oxidation of very long chain fatty acids and pristanic acid as well as C27 bile acids [70].

In 2006, Ferdinandusse et al. summarised the clinical and biochemical spectrum of D‐bifunctional protein deficiency [83]. Most affected individuals presented with neonatal hypotonia and seizures and died within the first 2 years without achieving any developmental milestones; however, 12 survived beyond 2 years and 5 beyond 7.5 years. Liver disease was present in a minority. There was a correlation between the extent of DHCA/THCA accumulation and survival. However, the major bile acids in plasma and bile can be varanic acid, 3α,7α,12α,24‐tetrahydroxy‐5β‐cholestanoic acid, and Δ24‐THCA is also present [70, 84, 85]. In urine, hydroxylated derivatives of Δ24‐THCA are detected [37, 70]. Little is known about the potential toxicity of Δ24‐THCA or varanic acid.

The mildest end of the spectrum of *HSD17B4* mutations is shown by three adult siblings with a slowly progressive, juvenile‐onset phenotype comprising cerebellar atrophy and ataxia, intellectual decline, hearing loss, hypogonadism, hyperreflexia, a demyelinating sensorimotor neuropathy, and (in two of three) supratentorial white matter changes [86].

There are no reports of bile acid replacement therapy in DBFP deficiency.

## 11 SCPX Thiolase Deficiency ( *SCP2* Pathogenic Variants)

In 2006, Ferdinandusse et al. described an individual with mutations in the gene encoding peroxisomal sterol carrier protein X, the thiolase that catalyses the final step of β‐oxidation of branched chain substrates including DHCA and THCA [87]. This was a 48‐year‐old man with a 28‐year history of dystonic head tremor and spasmodic torticollis; he had also had a problem with stuttering since 7 years and was diagnosed with hypergonadotrophic hypogonadism at age 29 years. Analysis of plasma showed raised pristanic acid and traces of DHCA and THCA. Analysis of urine showed the presence of abnormal bile alcohol glucuronides probably pentahydroxy‐27‐nor‐5β‐cholestan‐24‐ones and hexahydroxy‐27‐nor‐5β‐cholestan‐24‐ones formed by decarboxylation of 3α,7α,12α‐trihydroxy,24‐oxo‐5β‐cholestanoic acid, the substrate for the thiolase. No attempt at product replacement therapy was reported.

## 12 Bile Acid Amidation Defect: Bile Acid CoA: Amino Acid N‐Acyl Transferase Deficiency ( *BAAT* Pathogenic Variants)

In contrast to the other peroxisomal enzyme/transporter defects, in BAAT deficiency, there is no defect in the conversion of C27 bile acids to C24 bile acids; the defect is in the conversion of the CoA esters of CDCA and CA to their glycine and taurine conjugates, resulting in a build‐up of unconjugated bile acids and their sulphates and glucuronides.

Setchell et al. described 10 children with biochemical evidence of an amidation defect, 4 of whom had biallelic mutations in *BAAT* [88]. Their clinical presentation was with fat‐soluble vitamin malabsorption, and some showed growth failure and transient neonatal cholestatic hepatitis. Age at diagnosis ranged from 3 months to 14 years; symptoms were still present in the older children, suggesting that the fat‐soluble vitamin malabsorption and growth failure do not resolve spontaneously.

Treatment of five children with biallelic *BAAT* mutations with glycocholic acid (15 mg/kg/day) resulted in this conjugated bile acid becoming the main bile acid in duodenal bile with improved absorption of vitamin D2 and vitamin E [89]. Growth improved in 3/3 growth‐delayed prepubertal patients.

## 13 Peroxisome Biogenesis (Zellweger Spectrum) Disorders (Various *PEX* Gene Pathogenic Variants)

Disorders of peroxisome biogenesis lead to impaired function of many pathways including β‐oxidation of very long chain fatty acids, of tetracosa‐hexaenoic acid (to produce docosahexaenoic acid), of dicarboxylic acids, and of C27 bile acids, α‐oxidation followed by β‐oxidation of phytanic acid, ether phospholipid synthesis, glyoxalate detoxification, and L‐pipecolic metabolism. Systems affected include the brain (seizures, hypotonia, leukodystrophy), visual system (pigmentary retinopathy), auditory system (sensorineural deafness), kidney (polycystic disease) as well as the liver. Product replacement for the defect in β‐oxidation of C27 bile acids was never likely to be a holistic treatment for Zellweger spectrum disorders. However, as indicated above, there is evidence that hepatotoxicity in peroxisomal disorders is at least in part due to the 27 carbon bile acids DHCA and THCA (particularly in unconjugated form), so bile acid replacement might improve liver function by reducing the production of these C27 bile acids. Unfortunately, the individuals with the most severe liver involvement in ZSD tend to have elevated plasma levels of CA and CDCA and their conjugates, so there is a particular risk of toxicity of bile acid therapy.

In 1992, Setchell et al. reported treatment of a 6‐month‐old boy with ZSD with CDCA and CA (each at 23 mg/kg/day [90]). Over the next 8 months, there was a dramatic reduction in bilirubin and transaminases and the plasma levels and urinary excretions of cholestanoic acids. There were also improvements in weight gain, liver biopsy appearances, and neurological symptoms (reduction in seizures, increase in spontaneous movements, improved bladder function, and sucking and cough reflexes).

In 2016, Berendse et al. described bile acid replacement therapy treatment in 19 individuals with Zellweger spectrum disorders (genetically confirmed and with at least one of the following symptoms: elevated transaminases, growth retardation or neurological symptoms) [91]. Individuals with a life expectancy of < 1 year (the severe end of the Zellweger spectrum) were excluded. Patients were followed for a period of 2.5 years prior to onset of treatment to avoid the possibility that liver disease was improving spontaneously. Treatment was with CA 15 mg/kg/day increasing to 20 mg/kg/day if DHCA and THCA were still detectable in plasma and reducing to 10 mg/kg/day if the patient suffered diarrhoea or vomiting or a twofold rise in transaminases or conjugated bilirubin. Treatment reduced the plasma concentration and urinary excretion of C27 bile acids in most patients over the 9 months of the study. However, in the four individuals with advanced liver disease, CA treatment led to a rise in plasma transaminases, bilirubin and CA with only a minor reduction in C27 bile acids. When the study was extended for a further 12 months, Klouwer et al. found that the levels of C27 bile acids were still suppressed and rose significantly when CA treatment was discontinued; however, they found that there had been no significant changes in liver function tests, liver elasticity, coagulation parameters, fat soluble vitamin levels or body weight. They concluded that ‘no improvement of clinically relevant parameters was observed after 21 months of treatment’ [92].

In 2017, Heubi et al. published data on a cohort that included both single enzyme defects and Zellweger spectrum disorders (22 individuals) [44]. Both groups were treated with cholic acid (10–15 mg/kg/day). In the ZSD group, there was a reduction in the excretion of C27 bile acids, a reduction in plasma transaminases, and improved weight gain. The improvement was maintained on further follow‐up [93].

## 14 Conclusions With Regard to Peroxisomal Disorders

Ferdinandusse and Wanders suggested that, in peroxisomal disorders, hepatotoxicity is most likely when unconjugated C27 bile acids are at high levels in liver cells and that this is likely when there is a build‐up of their CoA esters in the cytosol. This does seem to be borne out by the fact that end‐stage liver disease has been described in a disorder of ABCD3, the peroxisomal THCA‐CoA importer, and some individuals with a peroxisome biogenesis disorder, while acyl‐CoA oxidase 2 deficiency tends to produce milder liver dysfunction, as do disorders of the steps beyond ACOX2. The fact that, in individuals with ACOX2 deficiency, abnormal liver function tests can improve with ursodeoxycholic acid treatment, which does not reduce synthesis of C27 bile acids, indicates clearly that treatment of the different peroxisomal disorders needs to be assessed as individual disorders; not all cases of trihydroxycholestanoic acidaemia are the same.

## 15 Lessons on Product Replacement Therapy From the Bile Acid Synthesis Disorders

In most cases of product replacement therapy, the aim is to achieve physiological and not supraphysiological levels of the replaced product. This is well illustrated by the treatment of bile acid synthesis disorders. As mentioned above, the taurine and glycine conjugates of CA and CDCA are potent detergents, and they cause necrosis of hepatocytes in patients with obstructive cholestasis [94]. Glycochenodeoxycholic is frequently mentioned as a major culprit; but in experimental animals with impaired function of the BSEP, CA feeding produces severe cholestatic liver disease [95]. So, with both CDCA and CA therapy, monitoring the levels of their conjugates in blood is advisable; high levels in blood suggest the likelihood that levels are also high in hepatocytes, and particularly if persistently high levels are being seen alongside a rise in transaminases, this indicates that the dose needs to be reduced.

Different blocks in a metabolic pathway can have very different effects. In the bile acid synthesis defects, there is considerable variation in the proportion of individuals with the disorder that develop clinically apparent liver disease in infancy, whether or not the usual natural history is for the liver disease to resolve spontaneously, whether individuals develop neurological disease later in life, and the nature of that neurological disease. It follows that clinical trials and follow‐up studies should not group together all inborn errors of bile acid metabolism. One of the most important considerations in the design of a clinical trial is that the study cohort is homogeneous.

Despite these lessons that have been learned, treatment of disorders of bile acid synthesis by product replacement is an example of a very successful intervention for some individuals for example reversing neuropsychiatric disease in CTX and normalisation of liver function parameters and prevention of progression of liver disease to end stage in 3β‐hydroxy‐Δ5‐C27‐steroid dehydrogenase deficiency.

## Author Contributions

P.T.C. produced a first draft of the manuscript. Corrections and additional material were provided by R.H. and E.M.

## Ethics Statement

This is a review, and the details of ethical approval can be found in the cited papers.

## Consent

The authors have nothing to report.

## Conflicts of Interest

P.T.C. has received funding for maintenance and improvement of bile acid analyses from Theravia who market cholic acid for the treatment of bile acid synthesis defects.

## Supporting information


**Data S1:** Supporting Information.

## Data Availability

Data sharing not applicable to this article as no datasets were generated or analysed during the current study.
