# Supplementary material for: Treatment of Inborn Errors by Product Replacement: The Example of Inborn Errors of Bile Acid Synthesis
Source: J Inherit Metab Dis. 2025 Aug 22;48(5):e70081. doi: 10.1002/jimd.70081 (PMC12374088; doi:10.1002/jimd.70081)
Supplement: Supplementary file 1 — Data S1: Supporting Information. [file JIMD-48-0-s001.pptx]

## Slide 1
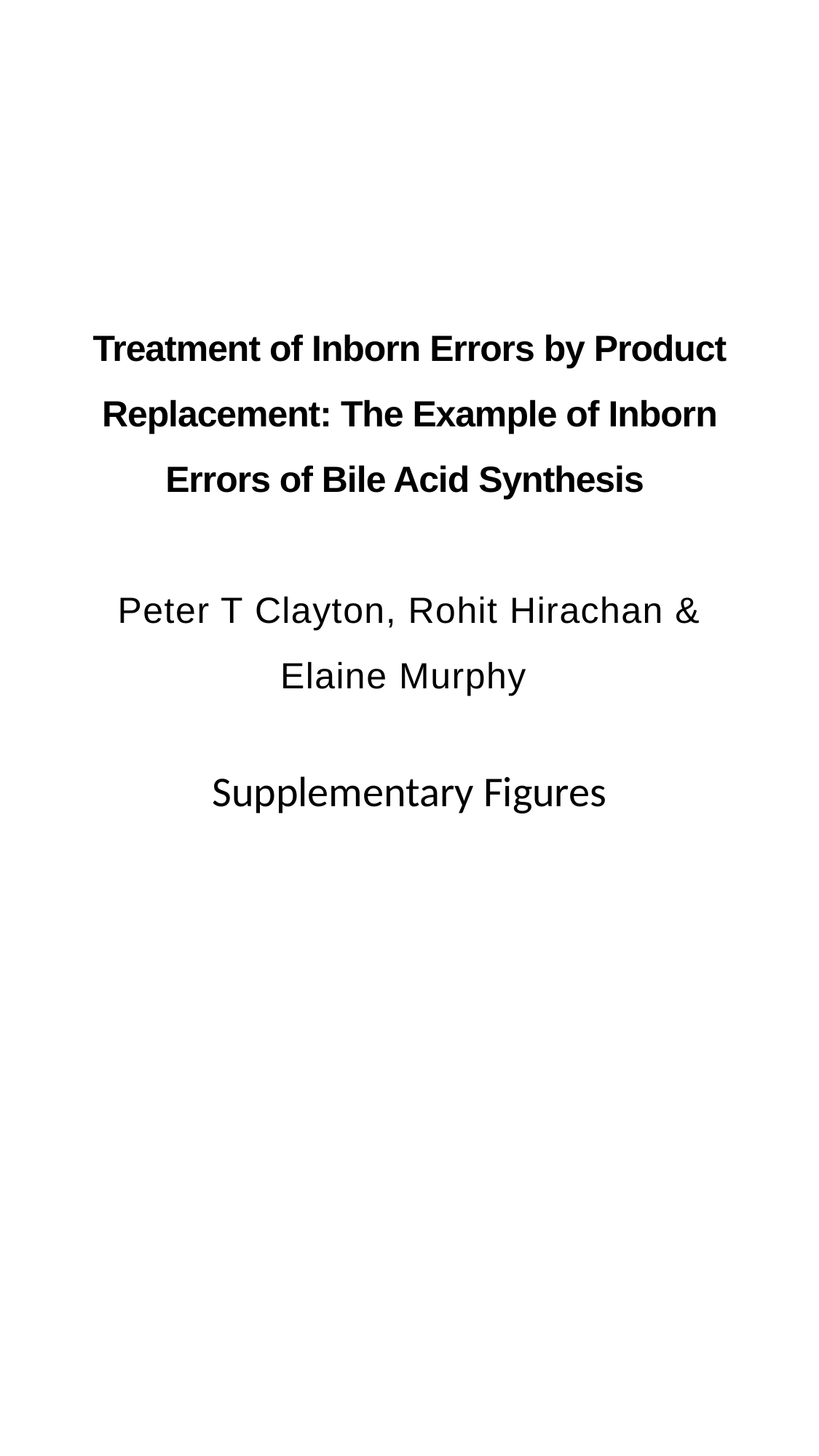

# Treatment of Inborn Errors by Product Replacement: The Example of Inborn Errors of Bile Acid Synthesis   Peter T Clayton, Rohit Hirachan & Elaine Murphy
Supplementary Figures

## Slide 2
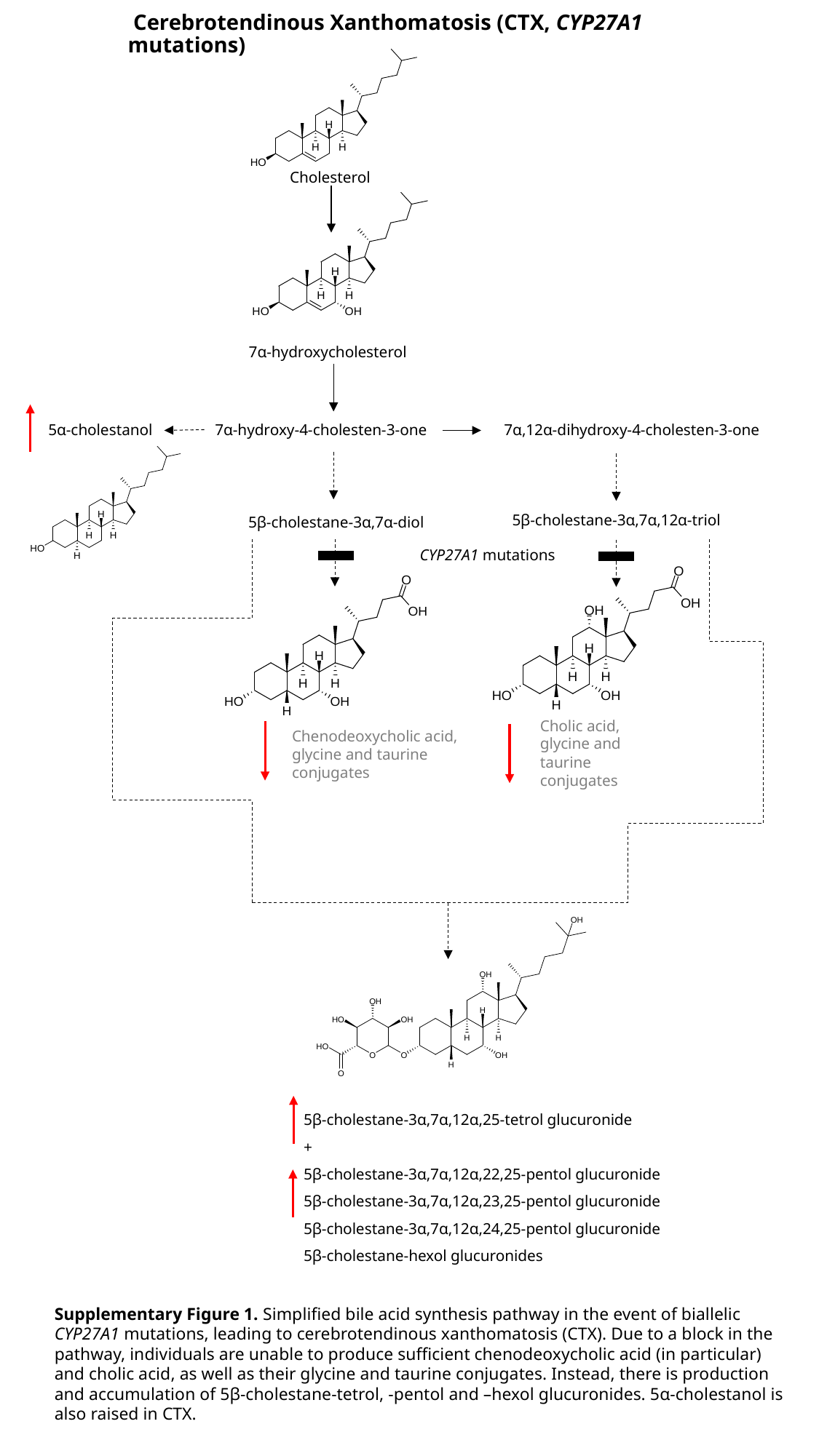

# Cerebrotendinous Xanthomatosis (CTX, CYP27A1 mutations)
Cholesterol
7α-hydroxycholesterol
5α-cholestanol
7α-hydroxy-4-cholesten-3-one
7α,12α-dihydroxy-4-cholesten-3-one
5β-cholestane-3α,7α,12α-triol
5β-cholestane-3α,7α-diol
CYP27A1 mutations
Cholic acid, glycine and taurine conjugates
Chenodeoxycholic acid, glycine and taurine conjugates
5β-cholestane-3α,7α,12α,25-tetrol glucuronide
+
5β-cholestane-3α,7α,12α,22,25-pentol glucuronide
5β-cholestane-3α,7α,12α,23,25-pentol glucuronide
5β-cholestane-3α,7α,12α,24,25-pentol glucuronide
5β-cholestane-hexol glucuronides
Supplementary Figure 1. Simplified bile acid synthesis pathway in the event of biallelic CYP27A1 mutations, leading to cerebrotendinous xanthomatosis (CTX). Due to a block in the pathway, individuals are unable to produce sufficient chenodeoxycholic acid (in particular) and cholic acid, as well as their glycine and taurine conjugates. Instead, there is production and accumulation of 5β-cholestane-tetrol, -pentol and –hexol glucuronides. 5α-cholestanol is also raised in CTX.

## Slide 3
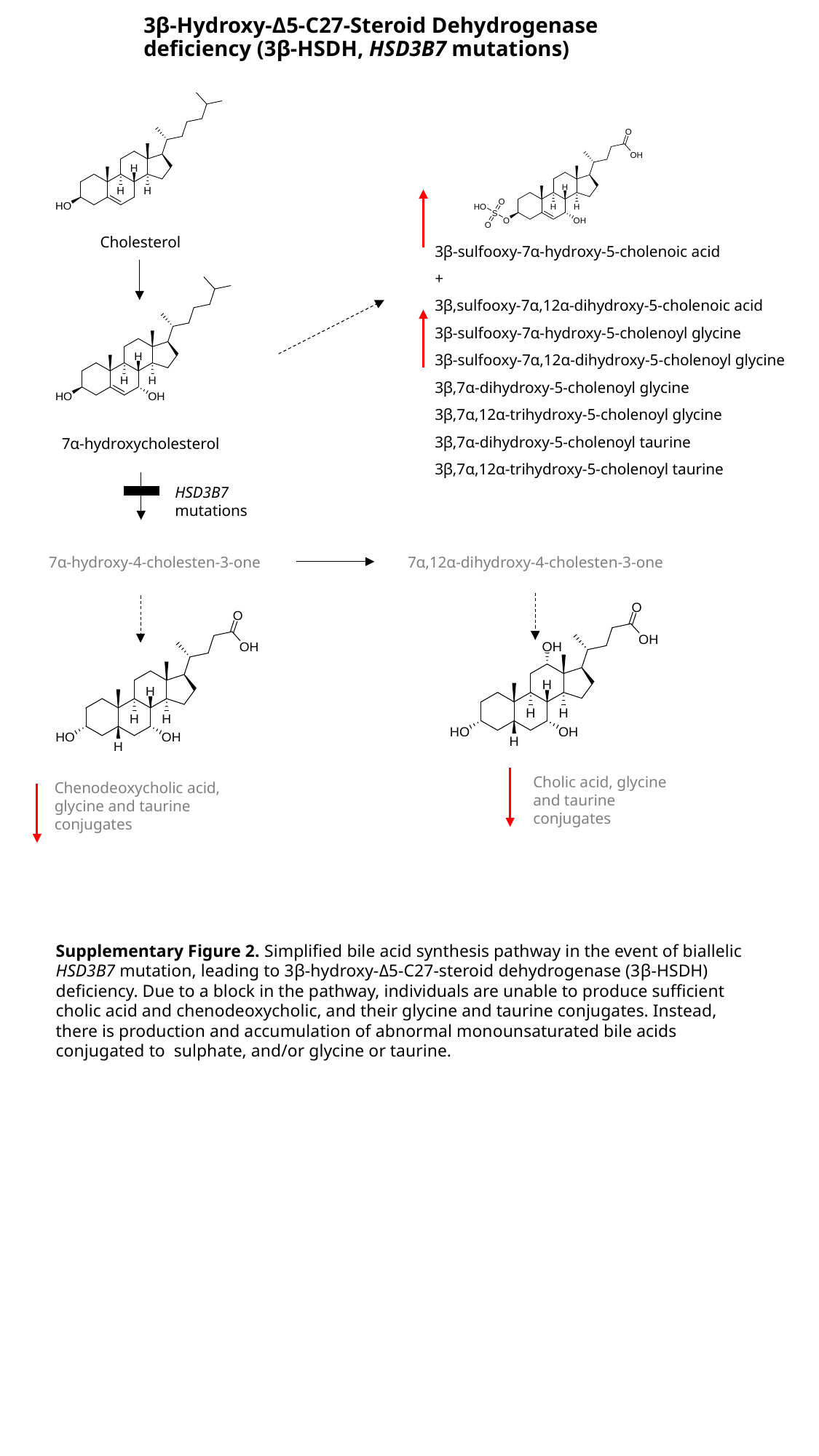

# 3β-Hydroxy-Δ5-C27-Steroid Dehydrogenase deficiency (3β-HSDH, HSD3B7 mutations)
Cholesterol
3β-sulfooxy-7α-hydroxy-5-cholenoic acid
+
3β,sulfooxy-7α,12α-dihydroxy-5-cholenoic acid
3β-sulfooxy-7α-hydroxy-5-cholenoyl glycine
3β-sulfooxy-7α,12α-dihydroxy-5-cholenoyl glycine
3β,7α-dihydroxy-5-cholenoyl glycine
3β,7α,12α-trihydroxy-5-cholenoyl glycine
3β,7α-dihydroxy-5-cholenoyl taurine
3β,7α,12α-trihydroxy-5-cholenoyl taurine
7α-hydroxycholesterol
HSD3B7 mutations
7α-hydroxy-4-cholesten-3-one
7α,12α-dihydroxy-4-cholesten-3-one
Cholic acid, glycine and taurine conjugates
Chenodeoxycholic acid, glycine and taurine conjugates
Supplementary Figure 2. Simplified bile acid synthesis pathway in the event of biallelic HSD3B7 mutation, leading to 3β-hydroxy-Δ5-C27-steroid dehydrogenase (3β-HSDH) deficiency. Due to a block in the pathway, individuals are unable to produce sufficient cholic acid and chenodeoxycholic, and their glycine and taurine conjugates. Instead, there is production and accumulation of abnormal monounsaturated bile acids conjugated to sulphate, and/or glycine or taurine.

## Slide 4
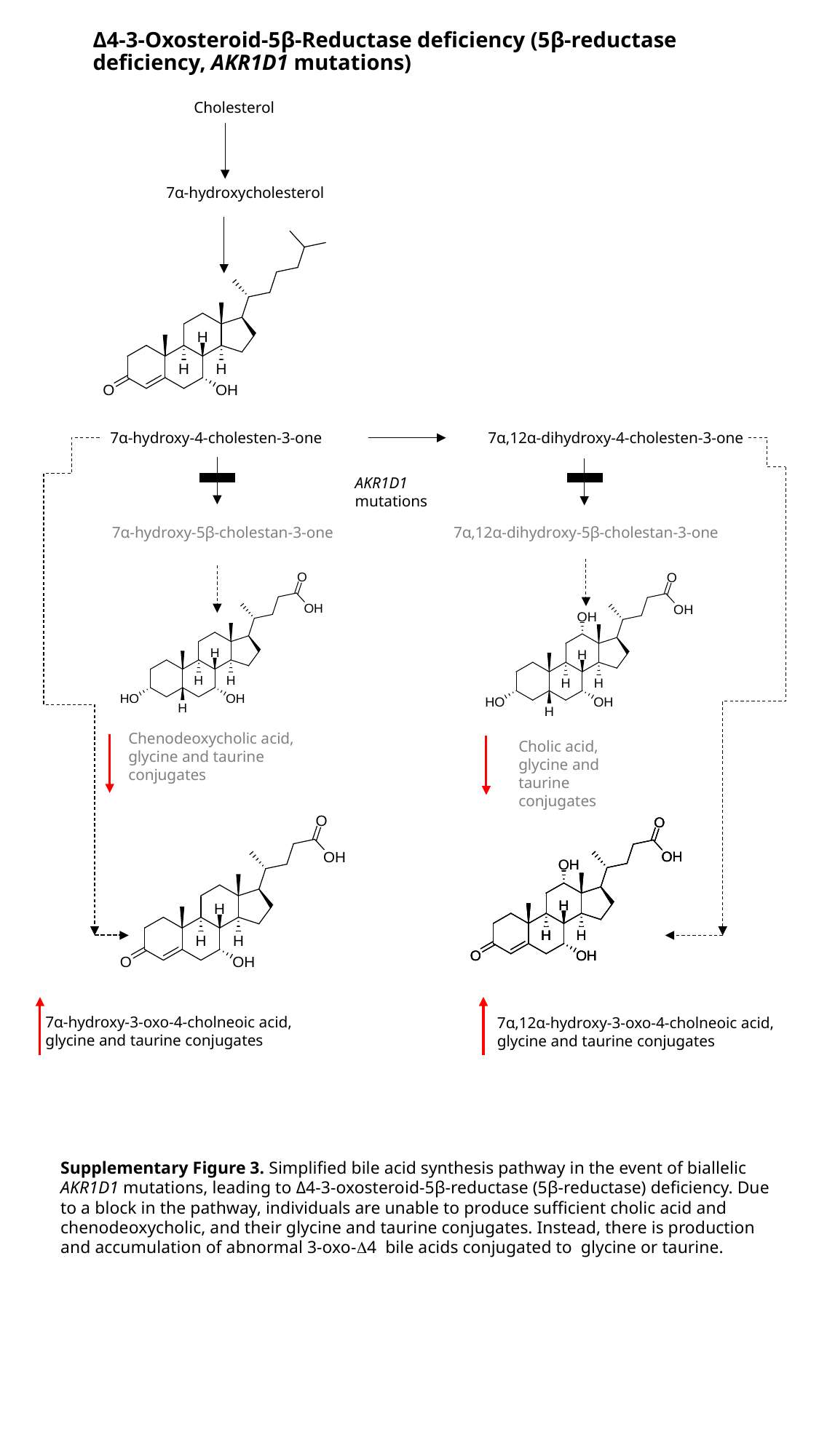

# Δ4-3-Oxosteroid-5β-Reductase deficiency (5β-reductase deficiency, AKR1D1 mutations)
Cholesterol
7α-hydroxycholesterol
7α,12α-dihydroxy-4-cholesten-3-one
7α-hydroxy-4-cholesten-3-one
AKR1D1 mutations
7α-hydroxy-5β-cholestan-3-one
7α,12α-dihydroxy-5β-cholestan-3-one
Chenodeoxycholic acid, glycine and taurine conjugates
Cholic acid, glycine and taurine conjugates
7α-hydroxy-3-oxo-4-cholneoic acid,
glycine and taurine conjugates
7α,12α-hydroxy-3-oxo-4-cholneoic acid,
glycine and taurine conjugates
Supplementary Figure 3. Simplified bile acid synthesis pathway in the event of biallelic AKR1D1 mutations, leading to Δ4-3-oxosteroid-5β-reductase (5β-reductase) deficiency. Due to a block in the pathway, individuals are unable to produce sufficient cholic acid and chenodeoxycholic, and their glycine and taurine conjugates. Instead, there is production and accumulation of abnormal 3-oxo-D4 bile acids conjugated to glycine or taurine.

## Slide 5
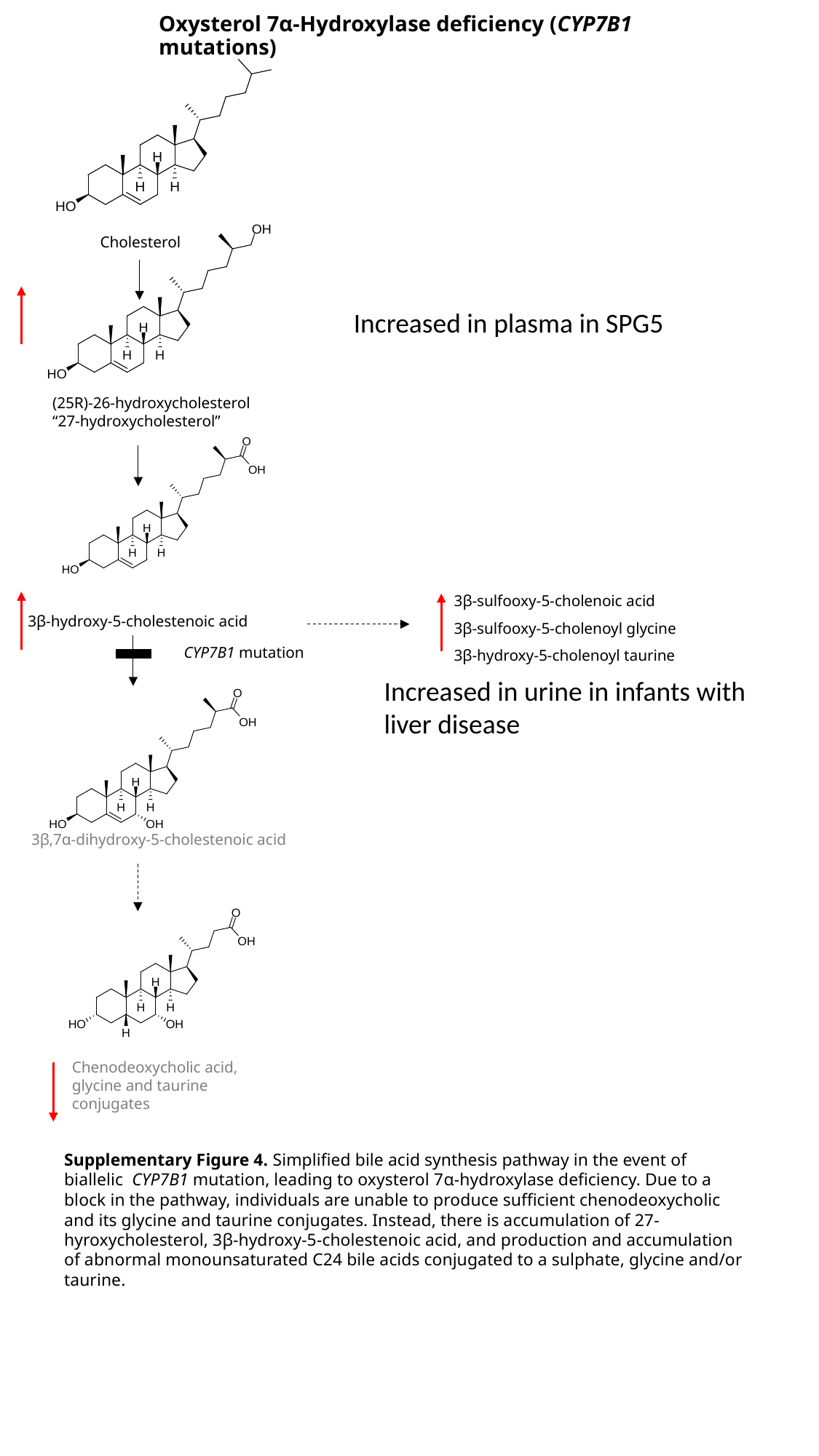

# Oxysterol 7α-Hydroxylase deficiency (CYP7B1 mutations)
Cholesterol
Increased in plasma in SPG5
(25R)-26-hydroxycholesterol
“27-hydroxycholesterol”
3β-sulfooxy-5-cholenoic acid
3β-sulfooxy-5-cholenoyl glycine
3β-hydroxy-5-cholenoyl taurine
3β-hydroxy-5-cholestenoic acid
CYP7B1 mutation
Increased in urine in infants with
liver disease
3β,7α-dihydroxy-5-cholestenoic acid
Chenodeoxycholic acid, glycine and taurine conjugates
Supplementary Figure 4. Simplified bile acid synthesis pathway in the event of biallelic CYP7B1 mutation, leading to oxysterol 7α-hydroxylase deficiency. Due to a block in the pathway, individuals are unable to produce sufficient chenodeoxycholic and its glycine and taurine conjugates. Instead, there is accumulation of 27-hyroxycholesterol, 3β-hydroxy-5-cholestenoic acid, and production and accumulation of abnormal monounsaturated C24 bile acids conjugated to a sulphate, glycine and/or taurine.

## Slide 6
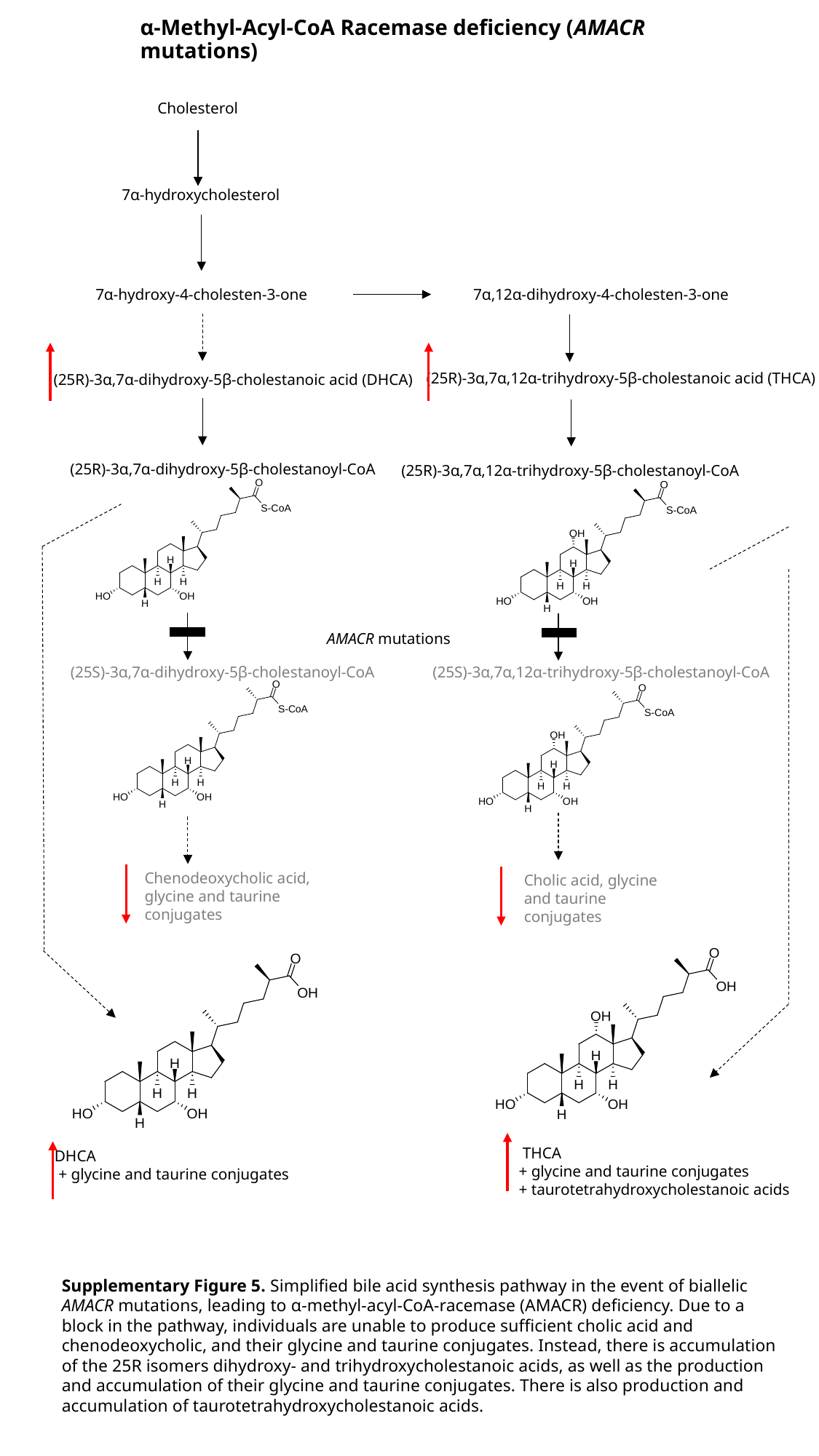

# α-Methyl-Acyl-CoA Racemase deficiency (AMACR mutations)
Cholesterol
7α-hydroxycholesterol
7α,12α-dihydroxy-4-cholesten-3-one
7α-hydroxy-4-cholesten-3-one
(25R)-3α,7α,12α-trihydroxy-5β-cholestanoic acid (THCA)
(25R)-3α,7α-dihydroxy-5β-cholestanoic acid (DHCA)
(25R)-3α,7α-dihydroxy-5β-cholestanoyl-CoA
(25R)-3α,7α,12α-trihydroxy-5β-cholestanoyl-CoA
AMACR mutations
(25S)-3α,7α-dihydroxy-5β-cholestanoyl-CoA
(25S)-3α,7α,12α-trihydroxy-5β-cholestanoyl-CoA
Chenodeoxycholic acid, glycine and taurine conjugates
Cholic acid, glycine and taurine conjugates
 THCA
+ glycine and taurine conjugates
+ taurotetrahydroxycholestanoic acids
DHCA
 + glycine and taurine conjugates
Supplementary Figure 5. Simplified bile acid synthesis pathway in the event of biallelic AMACR mutations, leading to α-methyl-acyl-CoA-racemase (AMACR) deficiency. Due to a block in the pathway, individuals are unable to produce sufficient cholic acid and chenodeoxycholic, and their glycine and taurine conjugates. Instead, there is accumulation of the 25R isomers dihydroxy- and trihydroxycholestanoic acids, as well as the production and accumulation of their glycine and taurine conjugates. There is also production and accumulation of taurotetrahydroxycholestanoic acids.

## Slide 7
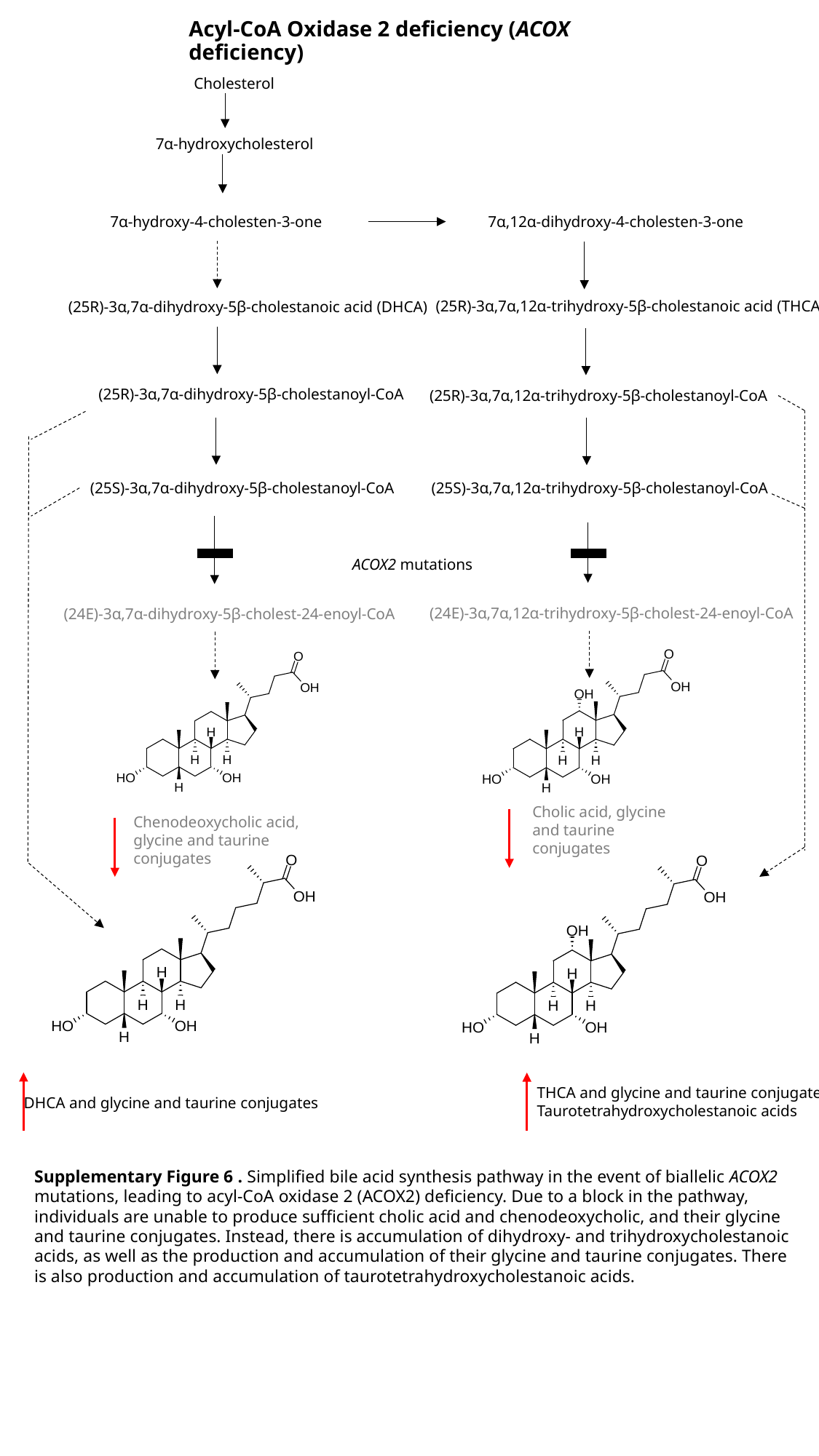

# Acyl-CoA Oxidase 2 deficiency (ACOX deficiency)
Cholesterol
7α-hydroxycholesterol
7α,12α-dihydroxy-4-cholesten-3-one
7α-hydroxy-4-cholesten-3-one
(25R)-3α,7α,12α-trihydroxy-5β-cholestanoic acid (THCA)
(25R)-3α,7α-dihydroxy-5β-cholestanoic acid (DHCA)
(25R)-3α,7α-dihydroxy-5β-cholestanoyl-CoA
(25R)-3α,7α,12α-trihydroxy-5β-cholestanoyl-CoA
(25S)-3α,7α,12α-trihydroxy-5β-cholestanoyl-CoA
(25S)-3α,7α-dihydroxy-5β-cholestanoyl-CoA
ACOX2 mutations
(24E)-3α,7α,12α-trihydroxy-5β-cholest-24-enoyl-CoA
(24E)-3α,7α-dihydroxy-5β-cholest-24-enoyl-CoA
Cholic acid, glycine and taurine conjugates
Chenodeoxycholic acid, glycine and taurine conjugates
THCA and glycine and taurine conjugated
Taurotetrahydroxycholestanoic acids
DHCA and glycine and taurine conjugates
Supplementary Figure 6 . Simplified bile acid synthesis pathway in the event of biallelic ACOX2 mutations, leading to acyl-CoA oxidase 2 (ACOX2) deficiency. Due to a block in the pathway, individuals are unable to produce sufficient cholic acid and chenodeoxycholic, and their glycine and taurine conjugates. Instead, there is accumulation of dihydroxy- and trihydroxycholestanoic acids, as well as the production and accumulation of their glycine and taurine conjugates. There is also production and accumulation of taurotetrahydroxycholestanoic acids.

## Slide 8
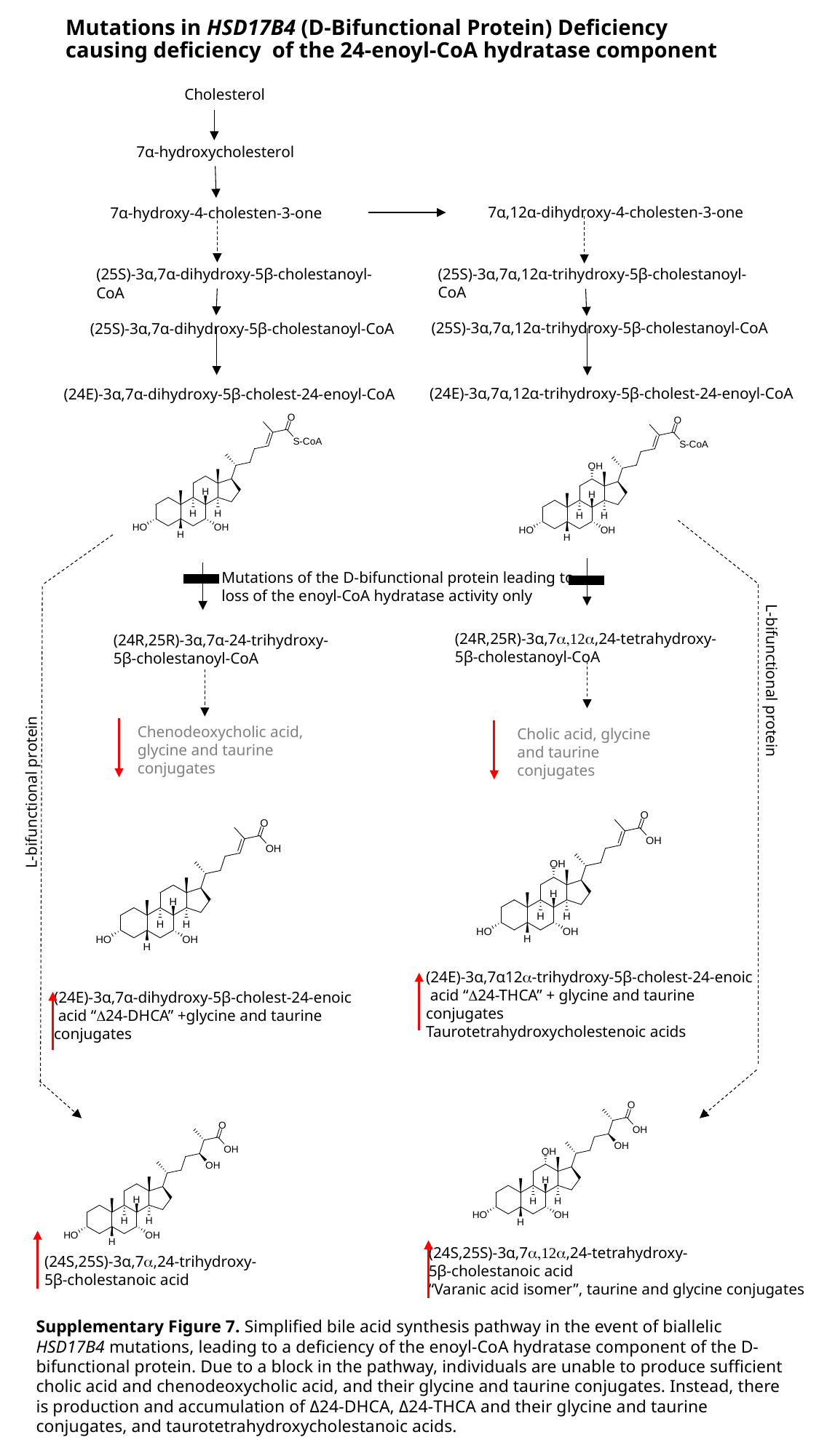

# Mutations in HSD17B4 (D-Bifunctional Protein) Deficiency causing deficiency of the 24-enoyl-CoA hydratase component
Cholesterol
7α-hydroxycholesterol
7α,12α-dihydroxy-4-cholesten-3-one
7α-hydroxy-4-cholesten-3-one
(25S)-3α,7α,12α-trihydroxy-5β-cholestanoyl-CoA
(25S)-3α,7α-dihydroxy-5β-cholestanoyl-CoA
(25S)-3α,7α,12α-trihydroxy-5β-cholestanoyl-CoA
(25S)-3α,7α-dihydroxy-5β-cholestanoyl-CoA
(24E)-3α,7α,12α-trihydroxy-5β-cholest-24-enoyl-CoA
(24E)-3α,7α-dihydroxy-5β-cholest-24-enoyl-CoA
Mutations of the D-bifunctional protein leading to
loss of the enoyl-CoA hydratase activity only
(24R,25R)-3α,7a,12a,24-tetrahydroxy-
5β-cholestanoyl-CoA
(24R,25R)-3α,7α-24-trihydroxy-
5β-cholestanoyl-CoA
L-bifunctional protein
Chenodeoxycholic acid, glycine and taurine conjugates
Cholic acid, glycine and taurine conjugates
L-bifunctional protein
(24E)-3α,7α12a-trihydroxy-5β-cholest-24-enoic
 acid “D24-THCA” + glycine and taurine
conjugates
Taurotetrahydroxycholestenoic acids
(24E)-3α,7α-dihydroxy-5β-cholest-24-enoic
 acid “D24-DHCA” +glycine and taurine
conjugates
(24S,25S)-3α,7a,12a,24-tetrahydroxy-
5β-cholestanoic acid
“Varanic acid isomer”, taurine and glycine conjugates
(24S,25S)-3α,7a,24-trihydroxy-
5β-cholestanoic acid
Supplementary Figure 7. Simplified bile acid synthesis pathway in the event of biallelic HSD17B4 mutations, leading to a deficiency of the enoyl-CoA hydratase component of the D-bifunctional protein. Due to a block in the pathway, individuals are unable to produce sufficient cholic acid and chenodeoxycholic acid, and their glycine and taurine conjugates. Instead, there is production and accumulation of Δ24-DHCA, Δ24-THCA and their glycine and taurine conjugates, and taurotetrahydroxycholestanoic acids.

## Slide 9
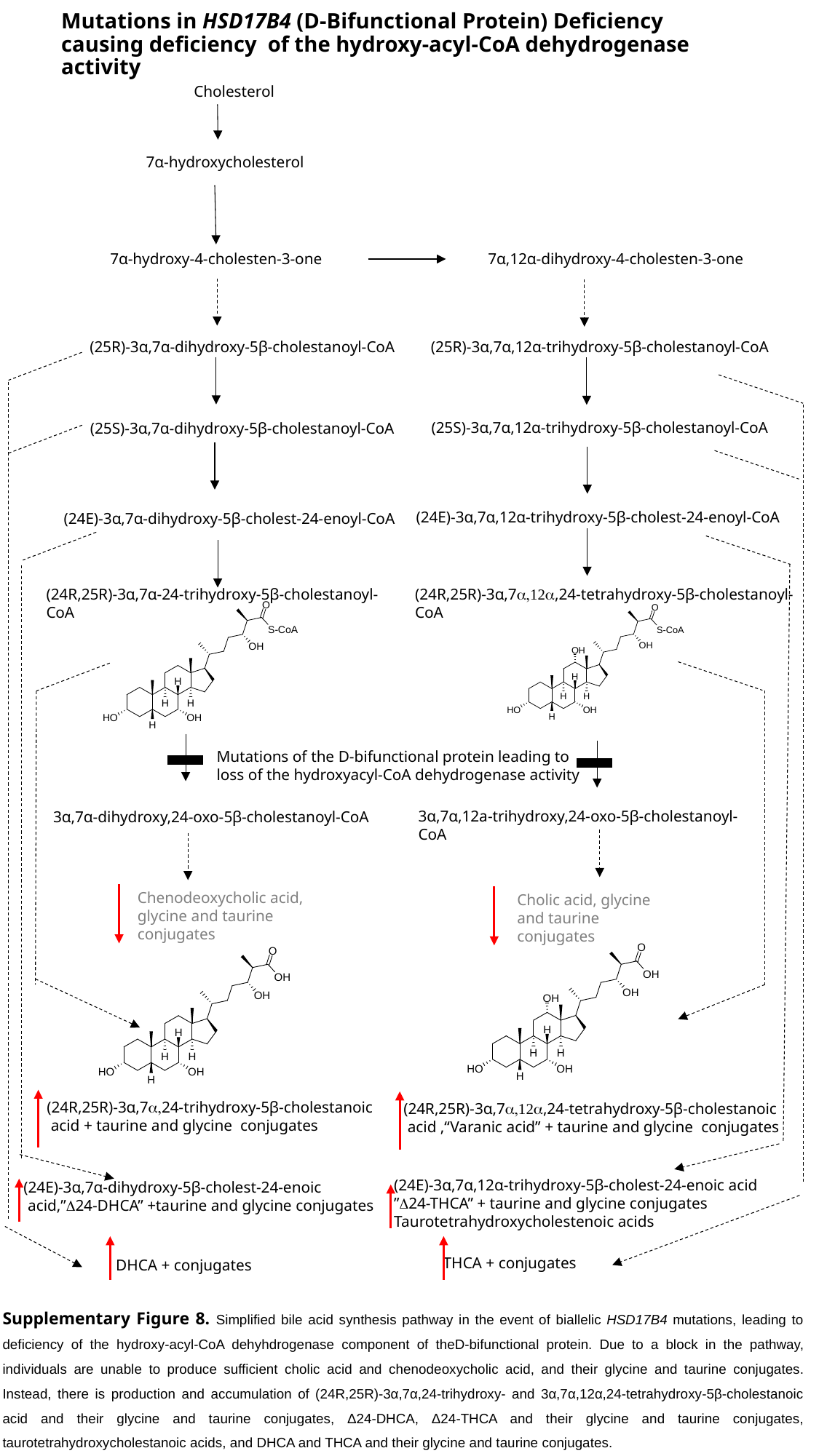

# Mutations in HSD17B4 (D-Bifunctional Protein) Deficiency causing deficiency of the hydroxy-acyl-CoA dehydrogenase activity
Cholesterol
7α-hydroxycholesterol
7α,12α-dihydroxy-4-cholesten-3-one
7α-hydroxy-4-cholesten-3-one
(25R)-3α,7α,12α-trihydroxy-5β-cholestanoyl-CoA
(25R)-3α,7α-dihydroxy-5β-cholestanoyl-CoA
(25S)-3α,7α,12α-trihydroxy-5β-cholestanoyl-CoA
(25S)-3α,7α-dihydroxy-5β-cholestanoyl-CoA
(24E)-3α,7α,12α-trihydroxy-5β-cholest-24-enoyl-CoA
(24E)-3α,7α-dihydroxy-5β-cholest-24-enoyl-CoA
(24R,25R)-3α,7α-24-trihydroxy-5β-cholestanoyl-CoA
(24R,25R)-3α,7a,12a,24-tetrahydroxy-5β-cholestanoyl-CoA
Mutations of the D-bifunctional protein leading to
loss of the hydroxyacyl-CoA dehydrogenase activity
3α,7α,12a-trihydroxy,24-oxo-5β-cholestanoyl-CoA
3α,7α-dihydroxy,24-oxo-5β-cholestanoyl-CoA
Chenodeoxycholic acid, glycine and taurine conjugates
Cholic acid, glycine and taurine conjugates
(24R,25R)-3α,7a,24-trihydroxy-5β-cholestanoic
 acid + taurine and glycine conjugates
(24R,25R)-3α,7a,12a,24-tetrahydroxy-5β-cholestanoic
 acid ,“Varanic acid” + taurine and glycine conjugates
(24E)-3α,7α,12α-trihydroxy-5β-cholest-24-enoic acid
”D24-THCA” + taurine and glycine conjugates
Taurotetrahydroxycholestenoic acids
(24E)-3α,7α-dihydroxy-5β-cholest-24-enoic
 acid,”D24-DHCA” +taurine and glycine conjugates
THCA + conjugates
DHCA + conjugates
Supplementary Figure 8. Simplified bile acid synthesis pathway in the event of biallelic HSD17B4 mutations, leading to deficiency of the hydroxy-acyl-CoA dehyhdrogenase component of theD-bifunctional protein. Due to a block in the pathway, individuals are unable to produce sufficient cholic acid and chenodeoxycholic acid, and their glycine and taurine conjugates. Instead, there is production and accumulation of (24R,25R)-3α,7α,24-trihydroxy- and 3α,7α,12α,24-tetrahydroxy-5β-cholestanoic acid and their glycine and taurine conjugates, Δ24-DHCA, Δ24-THCA and their glycine and taurine conjugates, taurotetrahydroxycholestanoic acids, and DHCA and THCA and their glycine and taurine conjugates.
